# Association between human herpesvirus infection and cervical carcinoma: a systematic review and meta-analysis

**DOI:** 10.1186/s12985-023-02234-5

**Published:** 2023-12-04

**Authors:** Han Zhang, Shunli Cai, Yuan Xia, Yangxuan Lin, Guozhong Zhou, Yinghui Yu, Min Feng

**Affiliations:** 1 Institute of Medical Biology, Chinese Academy of Medical Sciences and Peking Union Medical College, 935 Jiaoling Road, Kunming, 650118 China; 2 No.1 School of Clinical Medicine, Kunming Medical University, Kunming, 650051 China; 3 School of Basic Medical Sciences, Kunming Medical University, Kunming, 650051 China; 4 Department of Science and Research, The Affiliated Anning First People’s Hospital of Kunming University of Science and Technology, Kunming, Yunnan, 650302 China; 5 Department of Gynaecology and Obstetrics , The Affiliated Anning First People’s Hospital of Kunming University of Science and Technology, 2 Ganghe South Road, Anning City, Kunming, 650302 China

**Keywords:** Human herpesvirus, Cervical cancer, Precancerous cervical lesions, Meta-analysis, Herpes simplex virus type 2, Epstein-Barr virus

## Abstract

**Background:**

Cervical cancer (CC) is one of the most common gynecologic tumors among women around the world. Although the etiological role of human papillomavirus (HPV) in CC is well established, other factors in CC carcinogenesis remains unclear. Here, we performed a systematic review and meta-analysis to explore the association between infections of human herpesvirus (HHVs) and CC risk.

**Methods:**

Embase and PubMed databases were utilized to search the relevant studies. The revised JBI Critical Appraisal Tool was used to assess the quality of the included studies. Prevalence and odds ratios (ORs) with 95% confidence intervals (CI) were calculated to evaluate the association between viral infection and CC or precancerous cervical lesions (PCL).

**Results:**

Totally 67 eligible studies involving 7 different HHVs were included in meta-analysis. We found an increased risk of CC or PCL that was associated with the overall infection of HHVs (CC, OR = 2.74, 95% CI 2.13–3.53; PCL, OR = 1.95, 95% CI 1.58–2.41). Subgroup analysis showed a trend towards positive correlations between herpes simplex virus type 2 (HSV-2) infection and CC (OR = 3.01, 95% CI 2.24 to 4.04) or PCL (OR = 2.14, 95% CI 1.55 to 2.96), and the same is true between Epstein-Barr virus (EBV) infection and CC (OR = 4.89, 95% CI 2.18 to 10.96) or PCL (OR = 3.55, 95% CI 2.52 to 5.00). However, for HSV-1 and cytomegalovirus (HCMV), there was no association between viral infection and CC or PCL. By contrast, the roles of HHV-6, HHV-7, and Kaposi sarcoma–associated herpesvirus (KSHV) in cervical lesions were unclear due to the limited number of studies.

**Conclusions:**

This study provided evidence that HHVs infection as a whole increase the risk of CC incidence. In addition, some types of HHVs such as EBV and HSV-2 may serve as potential targets in the development of new interventions or therapeutic strategies for cervical lesions.

**Supplementary Information:**

The online version contains supplementary material available at 10.1186/s12985-023-02234-5.

## Introduction

Cervical cancer (CC) is one of the most common gynecologic tumors among women around the world. Despite CC could have been prevented through HPV vaccination [1], screening tests, and other potent inhibitors (such as carrageenan [2]) of HPV infection, the disease burden remains high worldwide. In 2020, an estimated 604,000 cases were newly diagnosed worldwide according to the data from WHO [3]. Although the etiological role of human papillomavirus (HPV) in CC has been well recognized, more than 90% of HPV infections are cleared within 2 years [4]. Only those persistent infection with high-risk HPV can lead to cancer, indicating that HPV is necessary but not sufficient for carcinogenesis. In addition to HPV, other mucosally transmitted pathogens have been implicated in the development of CC [5, 6]. In this context, a better understanding of viral cofactors involved in malignancy and tumor progression is vital for the interventive and therapeutic development in CC.

Human herpesviruses (HHVs) are a family of DNA viruses including herpes simplex virus type 1 and 2 (HSV-1 and HSV-2), varicella zoster virus (VZV), Epstein-Barr virus (EBV), cytomegalovirus (HCMV), human herpesvirus 6 and 7 (HHV-6 and HHV-7), and Kaposi sarcoma–associated herpesvirus (KSHV). Like HPV, HHVs causatively linked to a spectrum of human sexually transmitted diseases. Infections of HHVs are usually asymptomatic but more likely to establish a lifelong persistent infection [7], leading to modulation of the host immune response, host genome instability, or malignant transformation in the extreme case [8]. Except for VZV, viral DNA or RNA of HHVs has been detected in exfoliated cells or tissues from CC or cervical intraepithelial neoplasia (CIN) lesions [9–12], suggesting that most of HHVs exist in cervical epithelial cells with a possible oncogenic role. Of these HHVs, HSV, EBV, and HCMV have been identified to have high correlation with abnormal cervical cytology [9–11].

HSV-1 and HSV-2 are historically associated with oral and genital herpes, respectively, however, HSV-1 infection in genital tract continues to increase with the changes in sexual practices in recent years [13]. In 1968, the possibility of HSV-2 as a causal agent for CC was first reported in the journal of *Science* [14]. Later on, some studies demonstrated that HSV-2 seropositive women have a significantly increased risk of developing CC [15–18], and HSV DNA was able to be detected in CC tissues [16, 18]. However, another prospective study further pinpointed that there is no association between HSV-2 seroconversions and the development of cervical neoplasia [19], making the role of HSV-2 in CC controversial. In contrast, EBV is a well-established oncogenic virus associated with various lymphomas and some epithelial carcinomas [20, 21]. Of note, there is a correlation between EBV infection and abnormal cervical cytology [10]; on one hand, the prevalence of EBV positivity increases with lesion severity [22]; on the other hand, CIN or CC occurs more often among EBV positive women than those without EBV infection [23]. In addition to HSV and EBV, HCMV is also implicated as a co-factor in HPV-related CC [11].

Despite the correlations described above, the roles of HHVs in HPV-related CC remains incompletely understood. In the current study, we conducted a systematic review and meta-analysis to elucidate the potential roles of HHVs as a whole in the development of CC. The association between CC and the individual herpesvirus, i.e., HSV-1, HSV-2, HCMV, and EBV, was also investigated. Additionally, the effects of the possible influencing factors on the primary outcomes including virus detection methods, specimen type, stage of the disease, and different regions divided by the human development index (HDI) [24] were included in this analysis.

## Method

This study was registered in the International Prospective Register of Systmactic Reviews database (CRD42022314073) and followed the Preferred Reporting Items for Systematic Review and Meta-Analyses (PRISMA) reporting guideline.

### Search strategy

We searched Embase and PubMed using Medical Subject Headings (MeSH) terms and “search terms” (as listed in the Supplementary Methods). The most recent search was done on September 16, 2023. We applied no date or language restrictions. The reference list of identified papers was manually checked for additional relevant articles.

### Study selection criteria

Studies meeting the following criteria were included. First, the participants were women with cervical lesions (pathologically confirmed) and women with normal cervix. Meanwhile, cervical lesions include CC and/or precancerous cervical lesions (PCL). Second, the detections of HHVs antigens or antibodies were performed in all participants. Third, studies reported prevalence of HHVs infections or addressed the adjusted odds ratio (OR) for the association between cervical lesions and HHVs.

The exclusion criteria were as follows: (1) studies that included participants with CC or PCL combined with other genital malignancies; (2) methods for viral detections without detailed descriptions, such as “manufacturer information”, “detection of targets”, or “performed as the manufacturer’s guidelines”; (3) detection of viral infection using lymphocytes immune responses to viral antigens; (4) studies that lacked a control group; (5) studies that were published as abstracts, letters, case reports, or reviews; (6) studies that were repeated research results.

### Data extraction

A preconceived and standardized form was used for data collection. Extracted information included: (1) authors and year of publication; (2) countries where the research was conducted; (3) population investigated (types of cervical lesions); (4) specimen type; (5) method for viral detection; (6) relevant findings: number of individuals with cervical lesions and /or herpesviruses infections; (7) the adjusted OR values and their corresponding 95% confidence interval (CI), if applicable. Two authors (Yuan Xia and Yangxuan Lin) independently conducted study selection and data extraction, and all extracted data were cross checked by the third and fourth author (Shunli Cai and Han Zhang). Disagreements were resolved through consensus.

### Quality assessment

We assessed study quality using the revised JBI Critical Appraisal Tools of 8 items (Supplementary Methods). Studies with at least seven “yes” scores were considered to be of high methodology quality, those with between four and six “yes” scores to be of moderate quality and those with less than four “yes” scores to be of low methodological quality. Three authors (Yuan Xia, Yangxuan Lin, and Shunli Cai) performed this evaluation independently and disagreement was resolved through consensus and discussion.

### Statistical analysis

We analyzed the results by the pooled prevalence and odds ratio (OR). In the primary analysis, we first studied the overall association between HHVs and CC or PCL. Then, we studied individual HHV in cervical lesions of the pooled prevalence and OR value. For 9 studies that related to HSV-2 and reported adjusted effect estimates, we also conducted meta-analysis to pool the adjusted estimates.

Subsequently, univariable and multivariable random effects meta-regression analyses were performed to investigate factors associated with OR values, as well as to explain interstudy heterogeneity. According to the results of meta-analysis and mete regression, we further performed subgroup analysis for HSV-2 and EBV by different stages of disease (CIN 1, CIN 2/3, and CC), viral detection methods, and different HDI regions. In addition, we also assessed the OR value of EBV in specimen types.

The GRADE (Grading of Recommendation, Assessment, Development, and Evaluations) tool was used to assess the quality of evidence of the primary outcome [25]. The evidence was assigned a GRADE rating of very low, low, moderate or high by employing the five GRADE rating down considerations (risk of bias, heterogeneity between studies, indirectness, risk of random errors, and publication bias) and 3 factors may lead to rating up. Additionally, in the GRADE approach, observation studies start as low-quality evidence.

All statistical analyses were conducted using R statistical software version 4.2.0. Random effects model was used to calculate the pooled results and 95% CI. Heterogeneity was assessed using the I² statistic. Forest plots were generated to visualize the study-specific effect sizes along with 95% CI. We assessed publication bias using Peters test. All p values were two-sided. A p value of less than 0.05 was considered to be significant.

## Results

### Search results

**Fig. 1:**
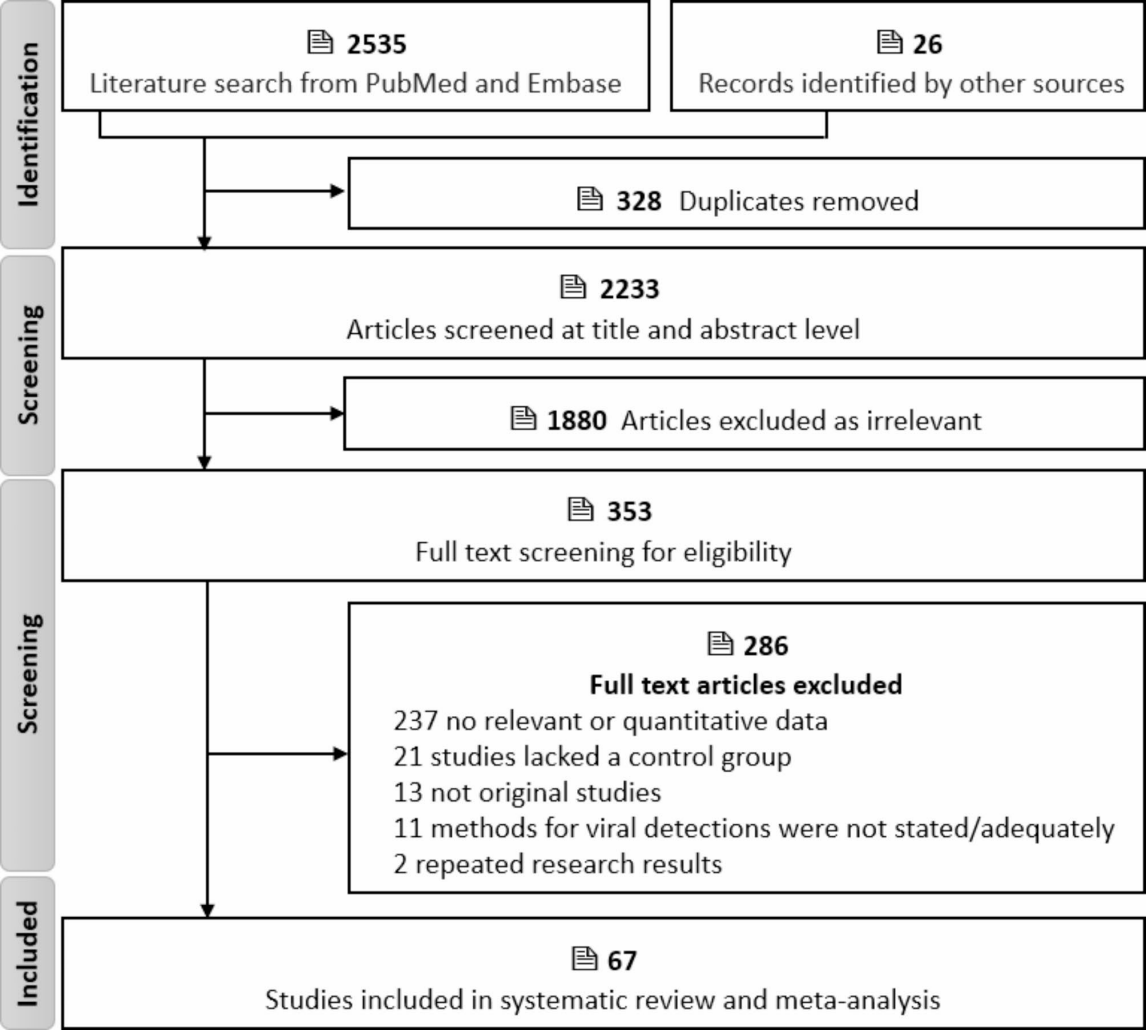
Preferred Reporting Items for Systematic Reviews and Meta-Analyses (PRISMA) flowchart

Totally 2233 publications were yielded after removal of duplicates, and 353 articles were left for full-text reading after excluding 1880 irrelevant records based on the screening of title and abstract. After full-text screening, 67 eligible publications [12, 15–19, 22, 23, 26–84] were included for the subsequent analysis (Fig. 1 and Table S1). Since 2 publications included data from a very high HDI country and a high HDI country, and 1 publication across 3 different HDI countries (very high, high and low), totally 71 studies were analyzed, including 2 studies in low HDI countries, 5 were in medium HDI countries, 19 were in high HDI countries, and 45 were in very high HDI countries. Viral nucleic acid was detected using PCR-based and hybridization-based assays in 25 and 7 studies, respectively. Virus-specific antibodies in serum were measured using immunological tests in 38 studies. Two different detection methods were used in 3 studies. The characteristics of the study are summarized in Table S1.

### Overall association between human herpesviruses (HHV) and CC or PCL

In all selected studies, the overall pooled prevalence of HHV among women with CC was 56% (95% CI 48–63%), whereas a significantly lower pooled proportion of 34% (95% CI 27–42%) was indicated in individuals with normal cervix (OR = 2.74, 95%CI 2.13 to 3.53) (Tables 1 and Fig. S1). Similarly, there was a significantly higher pooled prevalence (44%, 95% CI 35–54%) of HHV among women with PCL compared to normal women (28%, 95% CI 20–37%), the pooled OR was 1.95 (95% CI 1.58 to 2.41) (Tables 1 and Fig. S1).

### Association between individual HHV and CC or PCL

Seven different herpesviruses including HSV-1, HSV-2, HCMV, EBV, HHV-6, HHV-7, and KSHV were included in this review. The numbers of studies and individuals used to evaluate the association between CC or PCL were summarized in Table 1. The pooled prevalence and OR of viruses among patients with CC or PCL and corresponding controls were evaluated (Table 1). We failed to perform meta-analysis for HHV-6, HHV-7, and KSHV due to the small number of studies (2 or 3).

**Fig. 2:**
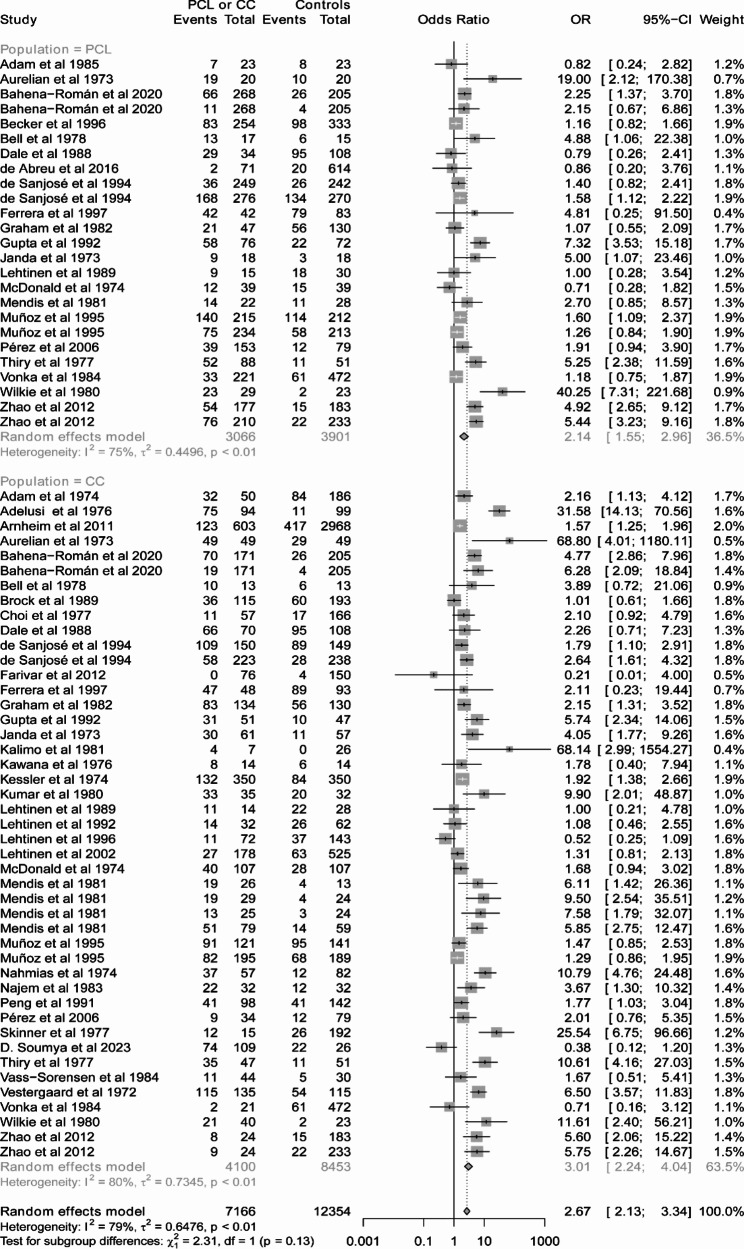
Forest plots of odds ratio for the association between HSV2 infection and cervical cancer (CC) or precancerous cervical lesions (PCL)

**Fig. 3:**
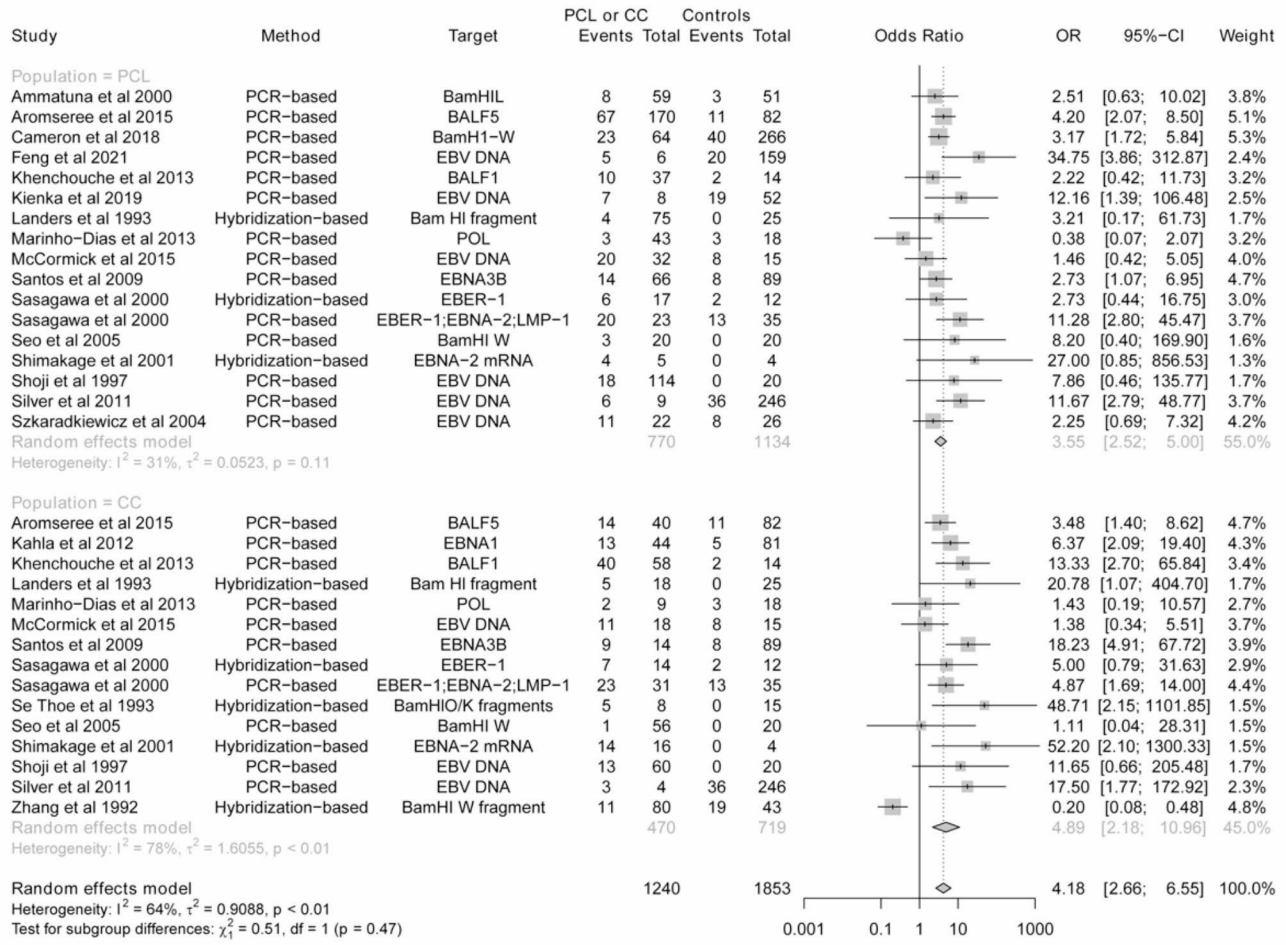
Forest plots of odds ratio for the association between EBV infection and cervical cancer (CC) or precancerous cervical lesions (PCL)

For HSV-1 and HCMV, there was no association between viral infection and CC or PCL (Tables 1 and Fig. S2-3). By contrast, there was an association between HSV-2 infection and CC (OR = 3.01, 95% CI 2.24 to 4.04), and the same is true between HSV-2 infection and PCL (OR = 2.14, 95% CI 1.55 to 2.96) (Fig. 2). The adjusted OR from 9 studies (Fig. S4 and Table S2) also indicated that HSV-2 could be a risk factor for CC (OR = 1.53, 95% CI 0.98 to 2.38) or PCL (OR = 2.53, 95% CI 1.28 to 4.99), although the 95% CI for OR of CC included 1. Lastly, we found an association between EBV infection and CC (OR = 4.89, 95% CI 2.18 to 10.96) or PCL (OR = 3.55, 95% CI 2.52 to 5.00) (Fig. 3).

**Table 1:** Pooled prevalence and OR of HHVs stratified by different stage of cervical lesion

Virus	Stage of disease	No of Studies	Total No of participants(case/control)	Cases	Control	Differences, χ^2^ test (P-value)	Random effects model OR (95% CI)	I^2^(%)
				Pooled prevalence of viral infection (%) (95%CI)	Pooled prevalence of viral infeciton (%) (95%CI)			
HHVs	PCL	42	6756/8326	44 (35–54)	28 (20–37)	0.01	1.95 (1.58–2.41)	66
	CC	55	5911/11,747	56 (48–63)	34 (27–42)	< 0.01	2.74 (2.13–3.53)	79
HSV-1	PCL	7	940/1513	44 (12–79)	47 (15–81)	0.91	1.06 (0.81–1.39)	0
	CC	11	609/1250	72 (48–91)	64 (42–83)	0.62	1.59 (0.73–3.45)	83
HSV-2	PCL	21	3066/3901	47 (34–61)	30 (20–41)	0.04	2.14 (1.55–2.96)	75
	CC	38	4100/8453	52 (44–60)	29 (22–36)	< 0.01	3.01 (2.24–4.04)	80
HCMV	PCL	7	1291/1202	55 (21–87)	50 (14–86)	0.86	1.30 (0.83–2.02)	28
	CC	6	611/932	67 (28–95)	59 (20–92)	0.78	1.46 (0.80–2.64)	41
EBV	PCL	16	770/1134	39 (25–54)	13 (6–21)	< 0.01	3.55 (2.52-5.00)	31
	CC	14	470/719	44 (29–59)	10(4–20)	< 0.01	4.89 (2.18–10.96)	78
HHV-6	PCL	2	199/190	33 (21–47)	11 (7–16)	< 0.01	NA	NA
	CC	1	30/7	33 (17–53)	0 (0–41)	< 0.01		
HHV-7	PCL	2	199/190	37 (0–90)	43 (7–83)	0.88	NA	NA
KSHV	PCL	2	291/800	10 (4–18)	10 (8–13)	0.86	NA	NA
	CC	2	91/386	17 (6–31)	12 (7–17)	0.45		

CC, cervical cancer; PCL, precancerous cervical lesions; OR, odds ratio

In addition, a small number of studies elucidate the presence of HHV-6, HHV-7 and KSHV in cervical samples. One study [36] from Italian women found that the prevalence of HHV-6 DNA was significantly higher in high-grade squamous intraepithelial lesions compared with normal women, whereas the prevalence of HHV-7 was low with no association with cervical lesions. Another study from Argentina [35] reported the similar results for HHV-6, but HHV-7 DNA was detected in all samples. Therefore, it seems that HHV-6 is a possible risk factor for cervical lesions. For KSHV, the positive rate for viral DNA was 8.7% in cervical biopsy samples from Chinese women with abnormal Papanicolaou smears [38]. However, no statistically significant association between KSHV and high-grade cervical lesions was found. Taken together, these findings suggest that genital tract is a possible transmission pathway for HHV-6, HHV-7, and KSHV, and their roles in cervical malignancy deserve further evaluation.

### Meta-regression and subgroup analysis of association between viral Infection and cervical lesions

The results of meta-regression are shown in Table S3-S7 and Table 2. We found that the risk of HSV-2 or EBV infection for cervical lesions varied according to viral detection methods, specimen types or different HDI regions, but not the year of publication. Furthermore, based on the above finding that HSV-2 and EBV infections are risk factors for CC or PCL, we performed subgroup analyses in terms of different stages of disease, viral detection methods, HDI regions, and specimen types (Table 2).

**Table 2:** Results of meta-regression and subgroup analysis of association between HSV-2 or EBV and cervical lesions

	HSV-2				EBV			
Analysis/subgroup	Studies (N)	OR (95% CI)	I^2^(%)	p value^a^	Studies (N)	OR (95% CI)	I^2^(%)	p value^a^
**Different stages of disease**								
CIN1	5	1.87 (0.95–3.70)	70	-	12	2.31 (1.54–3.47)	0	-
CIN2/3	10	1.64 (1.29–2.08)	48	-	14	4.32 (2.42–7.70)	38	-
CC	38	3.01 (2.24–4.04)	80	-	14	4.89(2.18–10.96)	78	-
**Viral detection methods**								
Immunological tests	36	2.41 (1.92–3.02)	77	0.0095	-	-	-	-
PCR-based	5	2.79 (1.79–4.34)	51	0.0187	15	3.92 (2.84–5.41)	36	0.7324
Hybridization-based	-	-	-	-	5	4.85(1.32–17.80)	76	Ref
**HDI regions**								
Low HDI	2	15.63 (3.18–76.90)	73	0.0116	-	-	-	-
Medium HDI	3	2.82 (1.05–7.54)	70	0.9988	2	6.70 (2.59–17.28)	51	0.3977
High HDI	7	2.84 (2.10–3.82)	70	Ref	8	3.53 (1.80–6.94)	78	Ref
Very high HDI	33	2.23 (1.74–2.85)	74	0.4365	9	3.65 (2.30–5.80)	9	0.6949
**Specimen types**								
Serum	36	2.50 (1.98,3.17)	79	0.0087	-	-	-	-
Brush/swab	5	3.34 (2.02,5.53)	58	0.0354	9	4.06 (2.37–6.95)	45	0.5600
Biopsy(fresh-frozen)	-	-	-	-	5	3.22 (1.24–8.33)	84	Ref
FFPE	-	-	-	-	4	6.61 (2.93–14.87)	0	0.1491

N, number; OR, odds ratio; Ref, reference

^a^ The p values were obtained through an univariate meta regression

According to the different stages of disease, results of subgroup analysis showed that HSV-2 was identified as a risk factor for CC (OR = 3.01, 95%CI 2.24–4.04) and CIN2/3 (OR = 1.64, 95%CI 1.29–2.08) except for CIN1 (OR = 1.87, 95%CI 0.95–3.70) (Tables 2 and Fig. S5A). Moreover, HSV-2 infection was associated with cervical lesions for studies using either immunological tests for detection of serum antibodies to HSV-2 (OR = 2.41, 95%CI 1.92–3.02) or PCR-based approaches for detection of genes encoding viral antigens (OR = 2.79, 95%CI 1.79–4.34) (Tables 2 and Fig. S5B). Testing for subgroup differences according to specimen types (Tables 2 and Fig. S5C) yielded the similar results as the subgroup analysis according to the viral detection methods (serum: OR = 2.50, 95%CI 1.98–3.17; brush/swab: OR = 3.34, 95%CI 2.02–5.53). We further analyzed the influence of different HDI regions on association between HSV-2 infection and cervical lesions (Tables 2 and Fig. S5D). The results showed a higher OR in low HDI countries (OR = 15.63, 95%CI 3.18–76.90), whereas lower OR values were found in medium, high, and very high HDI counties (medium: OR = 2.82, 95%CI 1.05–7.54; high: OR = 2.84, 95% CI 2.10–3.82; very high: OR = 2.23, 95% CI 1.74–2.85). In addition,

For EBV, regardless of the different stages of disease progression, subgroup analysis revealed an association of EBV infection and cervical lesions (Tables 2 and Fig. S6A). The pooled ORs were 2.31 (95%CI 1.54–3.47), 4.32 (95%CI 2.42–7.70), and 4.89 (95%CI 2.18–10.96) for CIN1, CIN2/3, and CC, respectively. In addition, we found that the studies using PCR- (OR = 3.92, 95%CI 2.84–5.41) and hybridization-based (OR = 4.85, 95%CI 1.32–17.80) assays showed significant relation between EBV infection and cervical lesions (Tables 2 and Fig. S6B). Further analysis regarding the different HDI regions showed a higher OR in medium HDI country (OR = 6.70, 95% CI 2.59–17.28), whereas lower OR values were found in high and very high HDI countries (high HDI: OR = 3.53, 95% CI 1.80–6.94; very high HDI: OR = 3.65, 95% CI 2.30–5.80) (Tables 2 and Fig. S6C). Lastly, we analyzed the effects of specimen types on association between EBV infection and CC or PCL. The results showed a higher OR in studies using formalin-fixed and paraffin-embedded (FFPE) samples (OR = 6.61, 95%CI 2.93–14.87), followed by studies using brush/swab (OR = 4.06, 95%CI 2.37–6.95) and biopsy (fresh-frozen) (OR = 3.22, 95% CI 1.24–8.33) samples (Tables 2 and Fig. S6D).

### GRADE assessment

We include five outcomes in the GRADE assessment: the associations between HHVs, HSV-1, HSV-2, HCMV or EBV infection and CC or PCL. We assessed the quality of evidence from ‘very low’ to ‘moderate’ for theses outcomes (Table S8).

### Publication bias

By using the Peters test, we did not find publication bias in HSV-1 (P = 0.7324), HCMV (P = 0.5436) and EBV (P = 0.7702), except for HSV-2 (P = 0.0036).

## Discussion

CC is the frequently occurring cancer of the female genital tract, and HPV infection is an established cause of CC. Other than HPV, the association between other viruses such as HHV and the risk of CC remains unclear. Therefore, it is of particularly necessary to perform this meta-analysis and systematic review. Based on our analyses, we found that the pooled prevalence of HHVs among CC or PCL patients are significantly higher than normal controls, suggesting that HHVs infections are very likely to increase the risk of cervical lesions. We also conducted four meta-analyses to explore the roles of HSV-1, HSV-2, HCMV, and EBV in cervical lesions, respectively. The results showed a trend towards a positive correlation between HSV-2 or EBV infections and cervical lesions, but there is no association between HSV-1 or HCMV and cervical lesions.

As one of the most common pathogens of sexually transmitted infection, HSV-2 was shown to be a risk factor for CC (OR = 3.01, 95% CI 2.24 to 4.04) and PCL (OR = 2.14, 95% CI 1.55 to 2.96) in the present study. Actually, the association between HSV-2 and CC has been debated for a long time. One meta-analysis [19] of longitudinal studies conducted in 2002 reported that HSV-2 was not associated with the risk of CC, but this study did not follow the Meta-analysis of Observational Studies in Epidemiology Guidelines [85]. In 2014, another meta-analysis [9] revealed an association between HSV-2 infection and CC in traditional case-control studies but not in nested case-control studies. Although the nested case-control study provides a high level of evidence, the number of such studies is relatively small. Given the fact that the small number of studies and participants may have impact on the validity of the results, we included both traditional and nested case-control studies, i.e., 38 studies enrolling 3991 CC patients and 8427 control individuals. Furthermore, the pooled adjusted OR estimates from 9 studies (adjustment for multiple factors including age, HPV status, number of sexual partners, et al.) also revealed the association between HSV-2 infection and CC or/and PCL (Fig. S4). In fact, it is hard to determine whether HSV-2 infection occurs simultaneously along with carcinogenesis due to the inability of serologic assays to distinguish the current infection from the past exposure of HSV-2. In this case, we performed subgroup analysis in terms of different viral detection methods. Indeed, similar results were obtained in both immunological tests for detection of serum antibodies to HSV-2 and viral DNA detection using PCR-based assays.

Moreover, we provided strong evidence that the incidence of CC is increased approximately 5-fold upon exposure to EBV, and the incidence of PCL is also increased upon EBV infection, in line with a previous meta-analysis [10] that showed a 4- and 2-times increase in the risk of CC and PCL incidence with EBV infection, respectively. Nonetheless, compared to the previous study [10], our meta-analysis included studies containing one or more control groups. On the other hand, we included more recent studies and performed more subgroup analyses. Of note, the subgroup analysis in terms of different stages of disease showed a positive correlation between the risk of EBV infection and lesion grade, further supporting the involvement of EBV in the development of CC. In addition, EBV detected with hybridization-based assays showed a higher pooled OR value than that using PCR-based assays, suggesting that EBV is a reliable cofactor in CC progression, since the former is the gold standard for EBV detection in tissues. To date, EBV infection in cervix is associated with an increased frequency of reactivation of EBV, viral shedding, and inflammation in the genital tract [86]. Previous studies suggested a potential cooperation of EBV with CC development by two possible mechanisms including synergizing with HPV and inducing local immunosuppression by infecting tissue-infiltrating lymphocytes [87]. Further elucidation of the mechanisms underlying the EBV-mediated tumorigenesis in CC is required.

Another interesting finding of our meta-analysis is that the risk of cervical lesions with HSV-2 or EBV infections negatively correlated to HDI distribution (Fig. S5D and Fig. S6C). For instance, the OR estimate for HSV-2 or EBV associated cervical lesions (included both CC and PCL) was obviously higher among low or medium HDI countries than high and very high HDI countries. According to these results, the women infected with HSV-2 or EBV in countries defined within the low ranking of the HDI are more likely to develop cervical lesions. This trend is also in agreement with the distribution of CC incidence worldwide [3]. One possible explanation is the unique socio-demographic characteristics of the lower HDI countries that might enhance the impact of HHVs infections on CC, which need to be taken into account in the future study.

Lastly, there are several limitations of this study. First, HPV infection is the main cause of CC, but the most studies in our analysis did not provide the data of HHVs and HPV co-infection. Thus, we failed to take the HPV infection into account in the subgroup analysis. Second, although we performed analyses in terms of different means of detection among studies, more detailed factors were not included in our analysis. For example, immunological tests for HSV-2 specific serum antibodies include ELISA, neutralization, complement fixation tests, radioimmunoassay, etc. In addition, different type of antibodies (IgA or IgG) with different cut-off values were applied. Thus, we cannot exclude the impacts of the above factors on the results.

Collectively, our results revealed the effects of HHVs infections on CC or PCL. We found a robust positive correlation between EBV infection and CC risk. Although the individual HHV-6, HHV-7, or KSHV was not independently analyzed, their potential roles in CC require further investigations. Importantly, our findings suggest HHVs (e.g., EBV or HSV-2) as potential targets in the development of new interventions or therapeutic strategies, including but not limited to vaccines and microbicides, for cervical lesions.

### Electronic supplementary material

Below is the link to the electronic supplementary material.

Supplementary Material 1

Supplementary Material 2

Supplementary Material 3

## Data Availability

The original contributions presented in the study are included in the article or Supplementary Material.
