# Supplementary material for: Association between human herpesvirus infection and cervical carcinoma: a systematic review and meta-analysis
Source: Virol J. 2023 Dec 4;20:288. doi: 10.1186/s12985-023-02234-5 (PMC10696706; doi:10.1186/s12985-023-02234-5)
Supplement: Supplementary file 1 — Supplementary Material 1 [file 12985_2023_2234_MOESM1_ESM.pdf]

Supplementary Methods

1. **Search strategy**

PubMed (the search yielded 961 publications)
#1("Uterine Cervical Neoplasms"[Mesh]) OR ((((((((((Cervical Neoplasm, Uterine) OR (Cervical Neoplasms, Uterine)) OR (Neoplasm, Uterine Cervical)) OR (Uterine Cervical Neoplasm)) OR (Neoplasms, Cervical)) OR (Cervical Neoplasm)) OR (Neoplasms, Cervix)) OR (Cervix Neoplasms)) OR (Cervical Cancer)) OR (Cervix Cancer)) OR (Cancer, Cervix))

#2 ("Cervical Intraepithelial Neoplasia"[Mesh]) OR ((((((Neoplasia, Cervical Intraepithelial) OR (Cervical Intraepithelial Neoplasms)) OR (Intraepithelial Neoplasm, Cervical)) OR (Neoplasm, Cervical Intraepithelial)) OR (Intraepithelial Neoplasia, Cervical)) OR (Cervical Intraepithelial Neoplasia, Grade III))

#3("Herpesvirus 1, Human"[Mesh]) OR ("herpes simplex virus 1"[Mesh]) OR ("Herpesvirus 2, Human"[Mesh]) OR ("herpes simplex virus 2"[Mesh]) OR ("Herpesvirus 3, Human"[Mesh]) OR ("Varicella Zoster virus"[Mesh]) OR ("Herpesvirus 4, Human"[Mesh]) OR ("Epstein-Barr virus"[Mesh]) OR ("Herpesvirus 5, Human"[Mesh]) OR ("Cytomegalovirus"[Mesh]) OR ("Herpesvirus 6, Human"[Mesh]) OR ("Herpesvirus 7, Human"[Mesh]) OR ("Herpesvirus 8, Human"[Mesh]) OR ("Kaposi sarcoma herpesvirus" [Mesh]) HHV OR HSV OR VZV OR EBV OR HCMV OR KSHV
#1 OR #2 AND #3

Embase (the search yielded 1359 publications)
#1'uterine cervix cancer'/exp OR 'uterine cervix cancer' OR 'neoplasms, cervical' OR 'neoplasms, cervix' OR 'cervical cancer'

#2'uterine cervix carcinoma in situ'/exp OR 'uterine cervix carcinoma in situ' OR 'cervical intraepithelial neoplasia' OR 'neoplasia, cervical intraepithelial' OR 'intraepithelial neoplasia, cervical'

#3 'human herpesvirus 1'/exp OR 'herpes simplex virus 1'/exp OR 'human herpes virus 2'/exp OR 'herpes simplex virus 2'/exp OR 'varicella zoster virus'/exp OR 'Epstein-Barr virus'/exp OR 'cytomegalovirus'/exp OR 'human herpesvirus 6'/exp OR 'human herpesvirus 7'/exp OR 'human herpesvirus 8'/exp OR 'Kaposi sarcoma herpesvirus'/exp OR HHV OR HSV OR EBV OR HCMV OR HBLV OR KSHV
#1 OR #2 AND #3

2. Quality assessment

The methodological quality of eligible studies was evaluated following the revised JBI Critical Appraisal Tools, which incorporates 8 items:

Q1: Were the criteria for inclusion in the sample clearly defined?
Q2: Were the study subjects and the setting described in detail?
Q3: Was the measurement of human herpesviruses infection in a valid and reliable way?
Q4: Were objective, standard criteria used for assessment of condition?
Q5: Were confounding factors identified?
Q6: Were strategies to deal with confounding factors stated?
Q7: Were the outcomes (cervical lesions) measured in a valid and reliable way?
Q8: Was appropriate statistical analysis used?

As recommended, for each included study, all criteria were scored as “yes,” “no,” or “unclear”. If an item was answered “no” or “unclear”, it was scored “0”; if the answer was “yes”, then scored “1”.
