# Supplementary material for: Association between human herpesvirus infection and cervical carcinoma: a systematic review and meta-analysis
Source: Virol J. 2023 Dec 4;20:288. doi: 10.1186/s12985-023-02234-5 (PMC10696706; doi:10.1186/s12985-023-02234-5)
Supplement: Supplementary file 2 — Supplementary Material 2 [file 12985_2023_2234_MOESM2_ESM.pdf]

Supplementary Table 1. Characteristics of eligible studies

Virus	Reference	Country	Population & Sample size	Method	Target	Type of sample	Score
HSV-2	Adam et al 1974	USA	CC = 50; CON = 186	Immunological (NT)	Antibodies	Serum	7
HSV-2	Adam et al 1985	USA	CIN = 23; CON = 23	Immunological (RIA)	Antibodies	Serum	6
HSV-2	Adelusi et al1976	Nigeria	CC = 94; CON = 99	Immunological (CF)	Antibodies	Serum	6
EBV	Ammatuna et al 2000	Italy	CIN1= 52; CIN2/3= 7; CON = 51	PCR-based (PCR)	BAMHI-L	Brush/swab	6
HHV-6	Arivananthan et al 1997	Malaysia	CC = 30; CON = 7	Hybridization-based (ISH)	HHV-6A/B	FFPE	6
HSV-2	Arnheim et al 2011	Finland et al	CC = 603; CON = 2968	Immunological (ELISA)	Antibodies	Serum	7
EBV	Aromseree et al 2015	Thailand	CC = 40; CIN1= 85;	PCR-based (PCR)	BALF5	Biopsy	5
			CIN2/3= 85; CON = 82;				
HSV-2	Aurelian et al 1973	USA	CC = 49; CON to CC = 49; PCL = 20; CON to PCL = 20	Immunological (NT)	Antibodies	Serum	6
HSV-2	Bahena-Román et al 2020	Mexico	CC = 171; CIN1 = 231; CIN2/3 = 37; CON = 205	Immunological (ELISA)	Antibodies	Serum	7
			CC = 171; CIN1 = 228; CIN2/3 = 40; CON = 205	PCR-based (RT-PCR)	HSV-2 DNA	Brush/swab	
HSV-1	Becker et al 1996	USA	CIN1 = 57; CIN2 = 74; CIN3 = 124; CON = 332	Immunological[a]	Antibodies	Serum	7
HSV-2			CIN1 = 57; CIN2 = 73; CIN3 = 124; CON = 333				
HSV-1/2	Bell et al 1978	USA	CC = 13; CON to CC = 13; PCL = 17; CON to PCL = 15	Immunological (NT)	Antibodies	Serum	6
HHV-6/7	Biganzoli et al 2020	Argentina	CIN1 = 61; CIN2/3 = 35; CON = 29	PCR-based (Nested PCR)	HHV-6/7 DNA	Brush/swab	7
HCMV	Broccolo et al 2008	Italy	CIN1 = 46; CIN2/3 = 57; CON = 66	PCR-based (RT-PCR)	HCMV DNA	Brush/swab	6
HHV-6/7					HHV-6/7 DNA		
HCMV	Brock et al 1989	Australia	CC = 115; CON = 193	Immunological	Antibodies	Serum	6
HSV-1/2							
EBV	Cameron et al 2018	Kenya	CIN1= 50; CIN2/3= 14; CON = 266	PCR-based (PCR)	BamH1-W	Brush/swab	7
HCMV	Chan et al 1999	China	CC = 30; CIN1 = 56; CIN2 = 34; CIN3 = 77; CON = 207	PCR-based (Nested PCR)	HCMV mtrII	Brush/swab	5
KSHV					ORF25; ORF26		
KSHV	Chavoshpour-Mamaghani et al 2021	Iran	Cervical cancer = 61; PCL = 124; CON = 179	PCR-based (PCR)	ORF26	FFPE<br>Brush/swab	7

HSV-2	Choi et al 1977	Canada	CC = 57; CON = 166	Immunological[b]	Antibodies	Serum	6
HSV-2	Dale et al 1988	UK	CC = 70; CIN = 34; CON = 108	Immunological (ELISA)	Antibodies	Serum	6
HCMV	Daxnerova et al 2003	USA	CIN1 = 154; CIN2/3 = 250; CON = 266	PCR-based (PCR)	HCMV DNA	Brush/swab	6
HSV-1/2	de Abreu et al 2016	Brazil	CIN2/3 = 71; CON = 614	PCR-based (PCR)	HSV-1/2 DNA	Brush/swab	7
HSV-2	de Sanjosé et al 1994	Spain	CC = 223; CON to CC = 238; CIN3 = 249; CON to CIN3 = 242	Immunological (ELISA)	Antibodies	Serum	7
HCMV		Colombia	CC = 150; CON to CC = 149; CIN3 = 276; CON to CIN3 = 270				
HSV-2	D.Soumya et al 2023	India	CC = 109; CON = 26	Immunological (ELISA)	Antibodies	Serum	6
HSV-2	Farivar et al 2012	Iran	CC = 76; CON = 150	PCR-based (RT-PCR)	HSV-2 DNA	FFPE	5
EBV	Feng et al 2021	China	CIN1 = 1; CIN2/3 = 5; CON = 159	PCR-based (RT-PCR)	EBV DNA	Brush/swab	6
HSV-2	Ferrera et al 1997	Honduras	CIN1 = 9; CON to CIN1 = 18; CIN2/3 = 33; CON to CIN2/3 = 65; CC = 48; CON to CC = 93;	Immunological (ELISA)	Antibodies	Serum	6
HCMV			CIN1 = 9; CON to CIN1 = 18; CIN2/3 = 32; CON to CIN2/3 = 63; CC = 49; CON to CC = 95				
HSV-2	Graham et al 1982	USA	CC = 134; PCL = 47; CON = 130	Immunological (RIA)	Antibodies	Serum	8
HSV-2	Gupta et al 1992	India	CC = 51; CON to CC = 47; CIN = 76; CON to CIN = 72	Immunological[c]	Antibodies	Serum	8
HSV-1/2	Janda et al 1973	Czechoslovakia	CC = 61; CON to CC= 57; PCL = 18; CON to PCL =18	Immunological (NT)	Antibodies	Serum	6
EBV	Kahla et al 2012	Tunisia	CC = 44; CON = 81	PCR-based (PCR)	EBNA1	Biopsy	8
HSV-2	Kalimo et al 1981	Finland	CC = 7; CON = 26	Immunological (RIA)	Antibodies[d]	Brush/swab	6
HSV-1/2	Kawana et al 1976	Japan	CC = 14; CON = 14	Immunological (CF)	Antibodies	Serum	6
HSV-2	Kessler et al 1974	Yugoslavia	CC = 350; CON = 350	Immunological (NT)	Antibodies	Serum	6
EBV	Khenchouche et al 2013	Algeria	CC = 58; CIN1 = 16; CIN2/3 = 21; CON = 14	PCR-based (PCR)	BALF1	Biopsy	6
HCMV	Kienka et al 2019	USA	CIN1= 4; CIN2/3= 4; CON = 52	PCR-based (RT-PCR)	HCMV DNA	Brush/swab	7
EBV					EBV DNA		
HSV-1/2	Kumar et al 1980	USA	CC = 35; CON = 32	Immunological (CF)	Antibodies	Serum	7
HCMV							
EBV	Landers et al 1993	Ireland	CC = 18; CIN1 = 25; CIN2/3 = 50; CON = 25	Hybridization-based (ISH)	BamH1 W	FFPE	6

HSV-2	Lehtinen et al 1989	Finland	CC = 14; CON to CC = 28; CIN3 = 15; CON to CIN3 = 30	Immunological (ELISA)	Antibodies	Serum	7
HSV-2	Lehtinen et al 1992	Finland	CC = 32; CON = 62	Immunological (ELISA)	Antibodies	Serum	6
HSV-2	Lehtinen et al 1996	Finland	CC = 72; CON = 143	Immunological (ELISA)	Antibodies	Serum	7
HSV-2	Lehtinen et al 2002	Norway et al	CC = 178; CON = 525	Immunological (ELISA)	Antibodies	Serum	7
EBV	Marinho-Dias et al 2013	Portugal	CC = 9; CIN1 = 21; CIN2/3 = 22; CON = 18	PCR-based (RT-PCR)	EBV POL	Brush/swab	6
HCMV							
EBV	McCormick et al 2015	Brazil	CC = 18; CIN1= 17; CIN2/3= 15; CON = 15	PCR-based (PCR)	EBV DNA	Brush/swab	5
HSV1/2	Mcdonald et al 1974	Canada	CC = 107; CON to CC = 107;	Immunological (NT)	Antibodies	Serum	4
			PCL = 39; CON to PCL = 39				
HSV-2	Mendis et al 1981	England	CC = 25; CON = 24	Immunological (ELISA)	Antibodies	Serum	7
		Sri Lanka	CC = 29; CON = 24				
		Malawi	CC = 26; CON = 13				
HSV-2	Mendis et al 1981	England	CC = 79; CON to CC = 59; PCL = 22; CON to PCL = 28	Immunological (ELISA)	Antibodies	Serum	5
HSV-2	Muñoz et al 1995	Spain	CC = 195; CON to CC = 189; CIN3 = 234; CON to CIN3 =213	Immunological (ELISA)	Antibodies	Serum	7
		Colombia	CC = 121; CON to CC = 141; CIN3 = 215; CON to CIN3 =212				
HSV-2	Nahmias et al 1974	USA	CC = 57; CON = 82	Immunological	Antibodies	Serum	7
HSV-1/2	Najem et al 1983	England	CC = 32; CON = 32	Immunological (ELISA)	Antibodies	Serum	4
HSV-2	Peng et al 1991	China	CC = 98; CON = 142	Immunological[e]	Antibodies	Serum	8
HSV-1/2	Pérez et al 2006	Argentina	CC = 34; CIN1 = 79; CIN2/3 = 74; CON = 79	PCR-based (PCR)	HSV-1/2 DNA	Brush/swab	7
EBV	Santos et al 2009	Brazil	CC = 14; CIN2/3 = 66; CON = 89	PCR-based (Nested PCR)	EBNA3B	Brush/swab	5
EBV	Sasagawa et al 2000	Japan	CC = 31; PCL = 23; CON = 35;	PCR-based[f]	EBV mRNA	Brush/swab	7
			CC = 14; PCL = 17; CON = 12	Hybridization-based (ISH)	EBER-1	FFPE	
EBV	Se Thoe et al 1993	Malaysia	CC = 8; CON = 15	Hybridization-based (ISH)	BamHlO/K	Biopsy	5
EBV	Seo et al 2005	Korea	CC = 56; CIN3 = 20; CON = 20	PCR-based (PCR)	BamHI-W	Brush/swab	5

EBV	Shimakage et al 2001	Japan	CC = 16; PCL = 5; CON = 4	Hybridization-based (ISH)	EBNA-2	FFPE	5
EBV	Shoji et al 1997	Japan	CC = 60; CIN1/2 = 44; CIN3 = 70; CON = 20	PCR-based (PCR)	EBV DNA	FFPE	6
EBV	Silver et al 2011	India	CC = 4; CIN1 = 2; CIN2/3 = 7; CON = 246	PCR-based (RT-PCR)	EBV DNA	Brush/swab	6
HSV-1/2	Skinner et al 1977	England	CC = 15; CON = 192	Immunological (CF)	Antibodies	Serum	6
EBV	Szkaradkiewicz et al 2004	Poland	CIN1/2 = 12; CIN3 = 10; CON = 26	PCR-based (PCR)	EBV DNA	Brush/swab	6
HSV-2	Thiry et al 1977	Belgium	CC = 47; PCL = 88; CON = 51	Immunological (NT)	Antibodies	Serum	5
HSV-2	Vass-Sorensen et al 1984	Norway	CC = 44; CON = 30	Immunological (Blotting)	Antibodies	Serum	6
HSV-1/2	Vestergaard et al 1972	Denmark	CC = 135; CON = 115	Immunological (CF)	Antibodies	Serum	6
HSV-2	Vonka et al 1984	Czechoslovakia	CC = 21; CIN2/3=221; CON = 472	Immunological (RIA)	Antibodies	Serum	5
HSV-2	Wilkie et al 1980	England	CC = 40; PCL = 29; CON = 23	Hybridization-based	HSV-2 RNA	Biopsy	5
EBV	Zhang et al 1992	China	CC = 80; CON = 43	Hybridization-based[g]	BamHI W	Biopsy	6
HSV-1/2	Zhao et al 2012	China	CC = 24; PCL = 210; CON = 233	PCR-based (RT-PCR)	HSV-1/2 DNA	Brush/swab	6
			CC = 24; PCL = 177; CON = 183	Immunological (ELISA)	Antibodies	Serum	

Note: CC = Cervical cancer; CON = Controls; PCL = Precancerous cervical lesion; NT = Neutralization test; RIA = Radioimmunoassay; CF = Complement fixation test; ISH = In situ hybridization; Biopsy = fresh-frozen tissue;

[a]purified glycoprotein assay; [b]indirect hemagglutination test; [c]indirect fluorescent antibody test; [d]cervical secretions; [e]western Blot; [f] reverse transcriptase-polymerase chain reaction; [g]southern blot.

References

Adam, E., Sanders, E. K., and Melnick, J. L. (1974). Antibodies to herpesvirus type 2 in breast cancer and cervical cancer patients. *Cancer* 33, 147-152.

Adam, E., Kaufman, R. H., and Adler-Storthz, K. (1985). A prospective study of association of herpes simplex virus and human papillomavirus infection with cervical neoplasia in women exposed to diethylstilbestrol in utero. *Int. J. Cancer* 35, 19-26.

Adelusi, B., Fabiyi, A., and Osunkoya, B. O. (1976). Herpes type 2 viruses and gynaecological malignancies. *International Journal of Gynecology and Obstetrics* 14, 209-212.

Ammatuna, P., Giovannelli, L., Giambelluca, D., Mancuso, S., Rubino, E., Colletti, P., et al. (2000). Presence of human papillomavirus and Epstein-Barr virus in the cervix of women infected with the human immunodeficiency virus. *J Med Virol* 62, 410-415.

Arivananthan, M., Yadav, M., and Kumar, S. (1997). Detection of HHV-6 genotypes by in situ hybridization with variant- specific oligonucleotide probes. *J. Virol. Methods* 66, 5-14.

Arnheim Dahlström, L., Andersson, K., Luostarinen, T., Thoresen, S., Ögmundsdottír, H., Tryggvadottír, L., et al. (2011). Prospective seroepidemiologic study of human papillomavirus and other risk factors in cervical cancer. *Cancer Epidemiol Biomarkers Prev* 20, 2541-2550.

Aromseree, S., Pientong, C., Swangphon, P., Chaiwongkot, A., Patarapadungkit, N., Kleebkaow, P., et al. (2015). Possible contributing role of Epstein-Barr virus (EBV) as a cofactor in human papillomavirus (HPV)-associated cervical carcinogenesis. *J. Clin. Virol.* 73, 70-76.

Aurelian, L., Davis, H. J., and Julian, C. G. (1973). Herpesvirus type 2 induced, tumor specific antigen in cervical carcinoma. *Am. J. Epidemiol.* 98, 1-9.

Bahena-Román, M., Sánchez-Alemán, M. A., Contreras-Ochoa, C. O., Lagunas-Martínez, A., Olamendi-Portugal, M., López-Estrada, G., et al. (2020). Prevalence of active infection by herpes simplex virus type 2 in patients with high-risk human papillomavirus infection: A cross-sectional study. *J Med Virol* 92, 1246-1252.

Becker, T. M., Lee, F., Daling, J. R., and Nahmias, A. J. (1996). Seroprevalence of and risk factors for antibodies to herpes simplex viruses, hepatitis B, and hepatitis C among southwestern Hispanic and non-Hispanic white women. *Sex Transm Dis* 23, 138-144.

Bell, R. B., Aurelian, L., and Cohen, G. H. (1978). Proteins of herpes virus type 2. IV. Leukocyte inhibition responses to type common antigen(s) in cervix cancer and recurrent herpetic infections. *Cell. Immunol.* 41, 86-102.

Biganzoli, P., Frutos, M. C., Venezuela, F., Mosmann, J., Kiguen, A., Pavan, J., et al. (2020). Detection of human herpesvirus 6 (HHV-6) and human herpesvirus 7 (HHV-7) DNA in endocervical samples from a positive and negative HPV woman of Córdoba, Argentina. *J Clin Pathol* 73, 30-34.

Broccolo, F., Cassina, G., Chiari, S., Garcia-Parra, R., Villa, A., Leone, B. E., et al. (2008). Frequency and clinical significance of human β-herpesviruses in cervical samples from Italian women. *J. Med. Virol.* 80, 147-153.

Brock, K. E., MacLennan, R., Brinton, J. L., Melnick, J. L., Adam, E., Mock, P. A., et al. (1989). Smoking and infectious agents and risk of in situ cervical cancer in Sydney, Australia. *Cancer Res.* 49, 4925-4928.

Cameron, J. E., Rositch, A. F., Vielot, N. A., Mugo, N. R., Kwatampora, J. K. L., Waweru, W., et al. (2018). Epstein-Barr Virus, High-Risk Human Papillomavirus and Abnormal

Cervical Cytology in a Prospective Cohort of African Female Sex Workers. *Sex Transm Dis* 45, 666-672.

Chan, P. K. S., Li, W. H., Chan, M. Y. M., and Cheng, A. F. B. (1999). Detection of human herpesvirus 8 in cervical cells of Chinese women with abnormal Papanicolaou smears. *Clin. Infect. Dis.* 29, 1584-1585.

Chavoshpour-Mamaghani, S., Shoja, Z., Mollaei-Kandelous, Y., Sharifian, K., and Jalilvand, S. (2021). The prevalence of human herpesvirus 8 in normal, premalignant, and malignant cervical samples of Iranian women. *Virol. J.* 18(1):144.

Choi, N. W., Shettigara, P. T., Abu-Zeid, H. A., and Nelson, N. A. (1977). Herpesvirus infection and cervical anaplasia: a seroepidemiological study. *Int J Cancer* 19, 167-171.

Dale, G. E., Coleman, R. M., Best, J. M., Benetato, B. B., Drew, N. C., Chinn, S., et al. (1988). Class-specific herpes simplex virus antibodies in sera and cervical secretions from patients with cervical neoplasia: a multi-group comparison. *Epidemiol Infect* 100, 445-465.

Daxnerova, Z., Berkova, Z., Kaufman, R. H., and Adam, E. (2003). Detection of human cytomegalovirus DNA in 986 women studied for human papillomavirus-associated cervical neoplasia. *J Low Genit Tract Dis* 7, 187-193.

de Abreu, A. L., Malaguti, N., Souza, R. P., Uchimura, N. S., Ferreira É, C., Pereira, M. W., et al. (2016). Association of human papillomavirus, Neisseria gonorrhoeae and Chlamydia trachomatis co-infections on the risk of high-grade squamous intraepithelial cervical lesion. *Am J Cancer Res* 6, 1371-1383.

de Sanjosé, S., Muñoz, N., Bosch, F. X., Reimann, K., Pedersen, N. S., Orfila, J., et al. (1994). Sexually transmitted agents and cervical neoplasia in Colombia and Spain. *Int J Cancer* 56, 358-363.

D. Soumya KM, M. Nagaraja, R. Rishi Gowtham, N. Umapathi, P. M. Madhavi Latha, A. M. Padmalatha, P. Prakash, Usha Kalawat: Detection of Chlamydia trachomatis and Herpes Simplex Virus-2 Infections Among Clinically Suspected Women with Cervical Cancer or Precancerous Lesions. Indian Journal of Gynecologic Oncology 2023, 21:1-7.

Farivar, T. N., Johari, P., Shafei, S., and Najafipour, R. (2012). Lack of association between herpes simplex virus type 2 infection and cervical cancer--Taq Man realtime PCR assay findings. *Asian Pacific journal of cancer prevention : APJCP* 13, 339-342.

Feng, M., Duan, R., Gao, Y., Zhang, H., Qiao, Y., Li, Q., et al. (2021). Role of Epstein-Barr Virus and Human Papillomavirus Coinfection in Cervical Intraepithelial Neoplasia in Chinese Women Living With HIV. *Front Cell Infect Microbiol* 11, 703259.

Ferrera, A., Baay, M. F., Herbrink, P., Figueroa, M., Velema, J. P., and Melchers, W. J. (1997). A sero-epidemiological study of the relationship between sexually transmitted agents and cervical cancer in Honduras. *Int J Cancer* 73, 781-785.

Graham, S., Rawls, W., Swanson, M., and McCurtis, J. (1982). Sex partners and herpes simplex virus type 2 in the epidemiology of cancer of the cervix. *Am J Epidemiol* 115, 729-735.

Gupta, M. M., Jain, R., Parashari, A., Singh, V., and Satyanarayana, L. (1992). HSV-IgA serum antibodies in cervical intraepithelial neoplasia and invasive cancer patients, and in their spouses: A case control study. *APMIS* 100, 598-604.

Janda, Z., Kanka, J., Vonka, V., and Svoboda, B. (1973). A study of herpes simplex type 2 antibody status in groups of patients with cervical neoplasia in Czechoslovakia. *Int. J. Cancer* 12, 626-630.

Kahla, S., Oueslati, S., Achour, M., Kochbati, L., Chanoufi, M. B., Maalej, M., et al. (2012). Correlation between ebv co-infection and HPV16 genome integrity in Tunisian cervical cancer patients. *Braz J Microbiol* 43, 744-753.

Kalimo, K., Terho, P., and Honkonen, E. (1981). Chlamydia trachomatis and herpes simplex virus IgA antibodies in cervical secretions of patients with cervical atypia. *British Journal of Obstetrics and Gynaecology* 88, 1130-1134.

Kawana, T., Cornish, J. D., Smith, M. F., and Aurelian, L. (1976). Frequency of antibody to a virus induced tumor associated antigen (AG 4) in Japanese sera from patients with cervical cancer and controls. *Cancer Res.* 36, 1910-1914.

Kessler, I. I., Kulcar, Z., and Rawls, W. E. (1974). Cervical cancer in Yugloslavia. I. Antibodies to genital herpesvirus in cases and controls. *Journal of the National Cancer Institute* 52, 369-376.

Khenchouche, A., Sadouki, N., Boudriche, A., Houali, K., Graba, A., Ooka, T., et al. (2013). Human papillomavirus and Epstein-Barr virus co-infection in cervical carcinoma in Algerian women. *Virol J* 10, 340.

Kienka, T., Varga, M. G., Caves, J., Smith, J. S., and Sivaraman, V. (2019). Epstein-Barr virus, but not human cytomegalovirus, is associated with a high-grade human papillomavirus-associated cervical lesions among women in North Carolina. *J Med Virol* 91, 450-456.

Kumar, A., Selim, M. S., Madden, D. L., Wallen, W. C., Vasquez, H. H., and Nankervis, G. A. (1980). Humoral-and cell-mediated immune responses to herpesvirus antigens in patients with cervical carcinoma. *Gynecol Oncol* 10, 18-25.

Landers, R. J., O'Leary, J. J., Crowley, M., Healy, I., Annis, P., Burke, L., et al. (1993). Epstein-Barr virus in normal, pre-malignant, and malignant lesions of the uterine cervix. *J Clin Pathol* 46, 931-935.

Lehtinen, M., Hakama, M., Knekt, P., Heinonen, P. K., Lehtinen, T., Paavonen, J., et al. (1989). Lack of serum antibodies to the major HSV-2 specified DNA-binding protein before diagnosis of cervical neoplasia. *J Med Virol* 27, 131-136.

Lehtinen, M., Hakama, M., Aaran, R. K., Aromaa, A., Knekt, P., Leinikki, P., et al. (1992). Herpes simplex virus type 2 infection and cervical cancer: a prospective study of 12 years of follow-up in Finland. *Cancer Causes Control* 3, 333-338.

Lehtinen, M., Dillner, J., Knekt, P., Luostarinen, T., Aromaa, A., Kirnbauer, R., et al. (1996). Serologically diagnosed infection with human papillomavirus type 16 and risk for subsequent development of cervical carcinoma: nested case-control study. *BMJ* 312, 537-539.

Lehtinen, M., Koskela, P., Jellum, E., Bloigu, A., Anttila, T., Hallmans, G., et al. (2002). Herpes simplex virus and risk of cervical cancer: a longitudinal, nested case-control study in the nordic countries. *Am. J. Epidemiol.* 156, 687-692.

Marinho-Dias, J., Ribeiro, J., Monteiro, P., Loureiro, J., Baldaque, I., Medeiros, R., et al. (2013). Characterization of cytomegalovirus and epstein-barr virus infection in cervical lesions in Portugal. *J. Med. Virol.* 85, 1409-1413.

McCormick, T. M., Canedo, N. H., Furtado, Y. L., Silveira, F. A., de Lima, R. J., Rosman, A. D., et al. (2015). Association between human papillomavirus and Epstein - Barr virus DNA and gene promoter methylation of RB1 and CDH1 in the cervical lesions: a transversal study. *Diagn Pathol* 10, 59.

McDonald, A. D., Williams, M. C., Manfreda, J., and West, R. (1974). Neutralizing antibodies to herpesvirus types 1 and 2 in carcinoma of the cervix, carcinoma in situ and cervical dysplasia. *Am. J. Epidemiol.* 100, 130-135.

Mendis, L. N., Best, J. M., and Senarath, L. (1981). A geographical study of antibodies to membrane antigens of HSV-2-infected cells and HSV-2-specific antibodies in patients with cervical cancer. *Int. J. Cancer* 28, 535-542.

Mendis, L. N., Best, J. M., and Banatvala, J. E. (1981). Class-specific antibodies (IgG and IgA) to membrane antigens of herpes simplex type-2 infected cells in patients with cervical dysplasia and neoplasia. *Int. J. Cancer* 27, 669-677.

Muñoz, N., Kato, I., Bosch, F. X., De Sanjosé, S., Sundquist, V. A., Izarzugaza, I., et al. (1995). Cervical cancer and herpes simplex virus type 2: case-control studies in Spain and Colombia, with special reference to immunoglobulin-G sub-classes. *Int. J. Cancer* 60, 438-442.

Nahmias, A. J., Naib, Z. M., and Josey, W. E. (1974). Epidemiological studies relating genital herpetic infection to cervical carcinoma. *Cancer Res.* 34, 1111-1117.

Najem, S. N., Vestergaard, B. F., and Potter, C. W. (1983). Herpes simplex virus type-specific antibodies detected by indirect and competition ELISA. Comparison of sera from patients with carcinoma of the uterine cervix, age matched controls and patients with recurrent genital herpes. *Acta Pathologica Microbiologica et Immunologica Scandinavica - Section B Microbiology* 91, 205-207.

Peng, H. Q., Liu, S. L., Mann, V., Rohan, T., and Rawls, W. (1991). Human papillomavirus types 16 and 33, herpes simplex virus type 2 and other risk factors for cervical cancer in Sichuan Province, China. *Int. J. Cancer* 47, 711-716.

Pérez, L. O., Barbisan, G., Abba, M. C., Laguens, R. M., Dulout, F. N., and Golijow, C. D. (2006). Herpes simplex virus and human papillomavirus infection in cervical disease in Argentine women. *Int. J. Gynecol. Pathol.* 25, 42-47.

Santos, N. B., Villanova, F. E., Andrade, P. M., Ribalta, J., Focchi, J., Otsuka, A. Y., et al. (2009). Epstein-Barr virus detection in invasive and pre-invasive lesions of the uterine cervix. *Oncol Rep* 21, 403-405.

Sasagawa, T., Shimakage, M., Nakamura, M., Sakaike, J., Ishikawa, H., and Inoue, M. (2000). Epstein-Barr virus (EBV) genes expression in cervical intraepithelial neoplasia and invasive cervical cancer: a comparative study with human papillomavirus (HPV) infection. *Hum Pathol* 31, 318-326.

Se Thoe, S. Y., Wong, K. K., Pathmanathan, R., Sam, C. K., Cheng, H. M., and Prasad, U. (1993). Elevated secretory IgA antibodies to Epstein-Barr virus (EBV) and presence of EBV DNA and EBV receptors in patients with cervical carcinoma. *Gynecol Oncol* 50, 168-172.

Seo, S. S., Kim, W. H., Song, Y. S., Kim, S. H., Kim, J. W., Park, N. H., et al. (2005). Epstein-Barr virus plays little role in cervical carcinogenesis in Korean women. *Int J Gynecol Cancer* 15, 312-318.

Shimakage, M., and Sasagawa, T. (2001). Detection of Epstein--Barr virus-determined nuclear antigen-2 mRNA by in situ hybridization. *J Virol Methods* 93, 23-32.

Shoji, Y., Saegusa, M., Takano, Y., Hashimura, M., and Okayasu, I. (1997). Detection of the Epstein-Barr virus genome in cervical neoplasia is closely related to the degree of infiltrating lymphoid cells: a polymerase chain reaction and in situ hybridization approach. *Pathol Int* 47, 507-511.

Silver, M. I., Paul, P., Sowjanya, P., Ramakrishna, G., Vedantham, H., Kalpana, B., et al. (2011). Shedding of epstein-barr virus and cytomegalovirus from the genital tract of women in a periurban community in Andhra Pradesh, India. *J. Clin. Microbiol.* 49, 2435-2439.

Skinner, G. R. B., Whitney, J. E., and Hartley, C. (1977). Prevalence of type specific antibody against type 1 and type 2 herpes simplex virus in women with abnormal cervical cytology; evidence towards pre pubertal vaccination of sero negative female subjects. *Arch. Virol.* 54, 211-221.

Szkaradkiewicz, A., Wal, M., Kuch, A., and Pieta, P. (2004). Human papillomavirus (HPV) and Epstein-Barr virus (EBV) cervical infections in women with normal and abnormal cytology. *Pol J Microbiol* 53, 95-99.

Thiry, L., Sprecher Goldberger, S., and Hannecart Pokorni, E. (1977). Specific non immunoglobulin G antibodies and cell mediated response to herpes simplex virus antigens in women with cervical carcinoma. *Cancer Res.* 37, 1301-1306.

Vass-Sorensen, M., Abeler, V., and Berle, E. (1984). Prevalence of antibodies to herpes simplex virus and frequency of HLA antigens in patients with preinvasive and invasive cervical cancer. *Gynecol. Oncol.* 18, 349-358.

Vestergaard, B. F., Hornsleth, A., and Pedersen, S. N. (1972). Occurrence of herpes- and adenovirus antibodies in patients with carcinoma of the cervix uteri. Measurement of antibodies to herpesvirus hominis (types 1 and 2), cytomegalovirus, EB-virus, and adenovirus. *Cancer* 30, 68-74.

Vonka, V., Kanka, J., and Hirsch, I. (1984). Prospective study on the relationship between cervical neoplasia and herpes simplex type-2 virus. II. Herpes simplex type-2 antibody presence in sera taken at enrolment. *Int. J. Cancer* 33, 61-66.

Wilkie, N. M., Eglin, R. P., Sanders, P. G., and Clements, J. B. (1980). The association of herpes simplex virus with squamous carcinoma of the cervix, and studies of the virus thymidine kinase gene. *Proceedings of the Royal Society of London - Biological Sciences* 210, 411-421.

Zhang, W., Jin, S. Q., Li, J. Y., Liang, X., Ming, L. H., Wang, X. H., et al. (1992). The infection of ebv for cervical epithelium-a new causitive agent in the development of cervical carcinomas? *Chin. J. Cancer Res.*, 26-32.

Zhao, Y., Cao, X., Zheng, Y., Tang, J., Cai, W., Wang, H., et al. (2012). Relationship between cervical disease and infection with human papillomavirus types 16 and 18, and herpes simplex virus 1 and 2. *J. Med. Virol.* 84, 1920-1927.

Supplementary Table 2. Summary of studies included in adjusted OR estimates

Study	Stage of disease	Adjusted odds ratio (95%CI)	Adjusted potential confounding factors
Arnheim et al 2011	CC	1.1 (0.9-1.5)	HPV16, HPV18, C. *trachomatis*, and cotinine
Ferrera et al 1997	CC	0.80 (0.04–15.1)	HPV
Lehtinen et al 1996	CC	0.6 (0.2-1.4)	Smoking and sexually transmitted disease
Muñoz et al 1995	PCL(CIN3)	1.17 (0.65-2.14)	age, center, parity, number of sexual partners
	CC	1.46 (0.82-2.59)	age at first sexual intercourse, education, smoking status, chlamydia antibodies and HPV status by PCR
Peng et al 1991	CC	1.3 (0.7-2.3)	age, income, residence, age at first marriage, and cigarette smoking
de Sanjosé et al 1994	PCL (CIN3)	1.2 (0.7-2.0)	age, center, number of sexual partners, age at first sex,
	CC	1.1 (0.6-1.7)	antibodies to C. *trachomatis*, HPV-PCR and males' partners
Gupta et al 1992	PCL (CIN1/2)	7.3 (2.3-22.2)	age
	PCL (CIN3)	8.9 (3.2-24.7)	
	CC	4.9 (2.0-12.2)	
Pérez et al 2006	PCL (CIN1)	1.7 (0.5–6.01)	age and HPV
	PCL (CIN2/3)	1.4 (0.4–4.7)	
	CC	1.6 (0.3–7.2)	
Zhao et al 2012	PCL (CIN)	4.9 (1.3-15.4)	age
	CC	4.7 (1.1-6.8)	

CC = cervical; PCL = precancerous cervical lesions; CIN = cervical intraepithelial neoplasia, grade 1 (CIN1), grade 1, grade 2 (CIN2), grade 3 (CIN3);

References

Arnheim Dahlström, L., Andersson, K., Luostarinen, T., Thoresen, S., Ögmundsdottír, H., Tryggvadottír, L., et al. (2011). Prospective seroepidemiologic study of human papillomavirus and other risk factors in cervical cancer. *Cancer Epidemiol Biomarkers Prev* 20, 2541-2550.

Ferrera, A., Baay, M. F., Herbrink, P., Figueroa, M., Velema, J. P., and Melchers, W. J. (1997). A sero-epidemiological study of the relationship between sexually transmitted agents and cervical cancer in Honduras. *Int J Cancer* 73, 781-785.

Lehtinen, M., Dillner, J., Knekt, P., Luostarinen, T., Aromaa, A., Kirnbauer, R., et al. (1996). Serologically diagnosed infection with human papillomavirus type 16 and risk for subsequent development of cervical carcinoma: nested case-control study. *BMJ* 312, 537-539.

Muñoz, N., Kato, I., Bosch, F. X., De Sanjosé, S., Sundquist, V. A., Izarzugaza, I., et al. (1995). Cervical cancer and herpes simplex virus type 2: case-control studies in Spain and Colombia, with special reference to immunoglobulin-G sub-classes. *Int. J. Cancer* 60, 438-442.

Peng, H. Q., Liu, S. L., Mann, V., Rohan, T., and Rawls, W. (1991). Human papillomavirus types 16 and 33, herpes simplex virus type 2 and other risk factors for cervical cancer in Sichuan Province, China. *Int. J. Cancer* 47, 711-716.

de Sanjosé, S., Muñoz, N., Bosch, F. X., Reimann, K., Pedersen, N. S., Orfila, J., et al. (1994). Sexually transmitted agents and cervical neoplasia in Colombia and Spain. *Int J Cancer* 56, 358-363.

Gupta, M. M., Jain, R., Parashari, A., Singh, V., and Satyanarayana, L. (1992). HSV-IgA serum antibodies in cervical intraepithelial neoplasia and invasive cancer patients, and in their spouses: A case control study. *APMIS* 100, 598-604.

Pérez, L. O., Barbisan, G., Abba, M. C., Laguens, R. M., Dulout, F. N., and Golijow, C. D. (2006). Herpes simplex virus and human papillomavirus infection in cervical disease in Argentine women. *Int. J. Gynecol. Pathol.* 25, 42-47.

Zhao, Y., Cao, X., Zheng, Y., Tang, J., Cai, W., Wang, H., et al. (2012). Relationship between cervical disease and infection with human papillomavirus types 16 and 18, and herpes simplex virus 1 and 2. *J. Med. Virol.* 84, 1920-1927.

Supplementary Table 3. Meta-regression analysis for association between HHVs and cervical lesions (PCL and CC)

	Univariate analysis			Multivariate analysis	
	p value	Estimate (95%CI)	R^2 (%)	p value	Estimate (95%CI)
Method			0.00		
Immunological	0.0357	-0.9204 (-1.7793 to -0.0616)		0.8127	0.1846 (-1.3423 to 1.7115)
PCR-based	0.0851	-0.7763 (-1.6599 to 0.1074)		0.9434	-0.0413 (-1.1823 to 1.0997)
Hybridization-based	Ref	Ref		Ref	Ref
Specimen			1.46		
Serum	0.0710	-0.6898 (-1.4386 to 0.0590)		0.1649	-1.0411 (-2.5106 to 0.4283)
FFPE	0.5257	0.3529 (-0.7370 to 1.4428)		0.6401	0.2676 (-0.8544 to 1.3897)
Brush/swab	0.0909	-0.6825 (-1.4737 to 0.1088)		0.1220	-0.7175 (-1.6268 to 0.1918)
Biopsy(fresh-frozen)	Ref	Ref		Ref	Ref
FFPE + Brush/swab	0.1449	-1.0789 (-2.5293 to 0.3716)		0.1604	-1.0534 (-2.5242 to 0.4175)
Biopsy + Brush/swab	0.1824	-1.1259 (-2.7809 to 0.5291)		0.1974	-1.1001 (-2.7730 to 0.5727)
HDI regions			8.57		
Low HDI	0.0057	1.9876 (0.5789 to 3.3962)		0.0027	2.2146 (0.7689 to 3.6604)
Medium HDI	0.4275	0.3013 (-0.4430 to 1.0456)		0.2401	0.4707 (-0.3146 to 1.2561)
High HDI	Ref	Ref		Ref	Ref
Very high HDI	0.9491	0.0121 (-0.3598 to 0.3840)		0.5931	0.1218 (-0.3250 to 0.5686)
Year of publication			0.11		
Before 2000	Ref	Ref		Ref	Ref
After 2001	0.4530	0.1350 (-0.2177 to 0.4877)		0.8333	0.0622 (-0.5167 to 0.6410)

Supplementary Table 4. Meta-regression analysis for association between HSV-1 and cervical lesions (PCL and CC)

	Univariate analysis			Multivariate analysis	
	p value	Estimate (95%CI)	R^2 (%)	p value	Estimate (95%CI)
Method			0.00		
Immunological	Ref	Ref		Ref	Ref
PCR-based	0.6247	-0.2814 (-1.4089 to 0.8461)		0.9235	0.1052 (-2.0427 to 2.2530)
Specimen			0.00		
Serum	0.3704	0.4496 (-0.5342 to 1.4333)		0.9102	-0.1848 (-3.3945 to 3.0249)
Brush/swab	Ref	Ref		Ref	Ref
HDI regions			0.00		
High HDI	Ref	Ref		Ref	Ref
Very high HDI	0.4056	0.4724 (-0.6410 to 1.5859)		0.8481	-0.2363 (-2.6551 to 2.1825)
Year of publication			0.00		
Before 2000	Ref	Ref		Ref	Ref
After 2001	0.3704	-0.4496 (-1.4333 to 0.5342)		0.4936	-1.0245 (-3.9578 to 1.9087)

Supplementary Table 5. Meta-regression analysis for association between HSV-2 and cervical lesions (PCL and CC)

	Univariate analysis			Multivariate analysis	
	p value	Estimate (95%CI)	R^2 (%)	p value	Estimate (95%CI)
Method			6.93		
Immunological	0.0095	-2.1160 (-3.7152 to -0.5169)		0.2865	0.6758 (-0.5671 to 1.9187)
PCR-based	0.0187	-2.0603 (-3.7783 to -0.3423)		0.0228	-2.0115 (-3.7425 to-0.2804)
Hybridization-based	Ref	Ref		Ref	Ref
Specimen			8.74		
Serum	0.0087	-2.1316 (-3.7235 to -0.5397)		0.0035	-2.9144 (-4.8732 to -0.9556)
FFPE	0.0138	-4.5944 (-8.2500 to -0.9387)		0.0206	-4.3065 (-7.9510 to -0.6620)
Brush/swab	0.0354	-1.8358 (-3.5459 to -0.1257)		0.0833	-1.5655 (-3.3370 to 0.2060)
Biopsy(fresh-frozen)	Ref	Ref		Ref	Ref
HDI regions			16.31		
Low HDI	0.0116	1.7478 (0.3910 to 3.1045)		0.0081	1.7998 (0.4670 to 3.1326)
Medium HDI	0.9988	0.0008 (-0.9876 to 0.9891)		0.7410	0.1650 (-0.8136 to 1.1437)
High HDI	Ref	Ref		Ref	Ref
Very high HDI	0.4365	-0.1978 (-0.6958 to 0.3003)		0.4292	-0.2169 (-0.7546 to 0.3208)
Year of publication			0.00		
Before 2000	Ref	Ref		Ref	Ref
After 2001	0.6624	-0.1208 (-0.6629 to 0.4214)		0.1649	-0.5021 (-1.2106 to 0.2065)

Supplementary Table 6. Meta-regression analysis for association between EBV and cervical lesions (PCL and CC)

	Univariate analysis			Multivariate analysis	
	p value	Estimate (95%CI)	R^2 (%)	p value	Estimate (95%CI)
Method			0.00		
PCR-based	0.7324	0.2032 (-0.9618 to 1.3683)		0.3582	0.6673 (-0.7562 to 2.0908)
Hybridization-based	Ref	Ref		Ref	Ref
Specimen			4.04		
FFPE	0.1491	0.9228 (-0.3309 to 2.1765)		0.0085	2.5882 (0.6617 to 4.5147)
Brush/swab	0.5600	0.3267 (-0.7718 to 1.4251)		0.2862	0.7933 (-0.6645 to 2.2511)
Biopsy(fresh-frozen)	Ref	Ref		Ref	Ref
Biopsy + Brush/swab	0.3951	-0.7844 (-2.5923 to 1.0235)		0.2160	-1.0417 (-2.6919 to 0.6084)
HDI regions			0.00		
Medium HDI	0.3977	0.6647 (-0.8758 to 2.2052)		0.7145	-0.3131 (-1.9905 to 1.3644)
High HDI	Ref	Ref		Ref	Ref
Very high HDI	0.6949	0.1959 (-0.7833 to 1.1751)		0.0608	-1.4516 (-2.9693 to 0.0661)
Year of publication			8.45		
Before 2000	Ref	Ref		Ref	Ref
After 2001	0.4247	0.5323 (-0.7745 to 1.8392)		0.4085	0.6383 (-0.8754 to 2.1520)

Supplementary Table 7. Meta-regression analysis for association between HCMV and cervical lesions (PCL and CC)

	Univariate analysis			Multivariate analysis	
	p value	Estimate (95%CI)	R^2 (%)	p value	Estimate (95%CI)
Method			0.00		
Immunological	Ref	Ref		Ref	Ref
PCR-based	0.4248	0.2692 (-0.3919 to 0.9302)		0.3894	0.8108 (-1.0357 to 2.6574)
Specimen			0.00		
Serum	0.4248	-0.2692 (-0.9302 to 0.3919)		0.3959	-0.7719 (-2.5541 to 1.0103)
Brush/swab	Ref	Ref		Ref	Ref
HDI regions			0.00		
Medium HDI	0.7717	-0.2240 (-1.7373 to 1.2893)		0.7506	0.3167 (-1.6366 to2.2701)
High HDI	Ref	Ref		Ref	Ref
Very high HDI	0.2430	0.5495 (-0.3729 to 1.4719)		0.2071	1.0128 (-0.5606 to 2.5862)
Year of publication			0.00		
Before 2000	Ref	Ref		Ref	Ref
After 2001	0.3089	0.3885 (-0.3599 to 1.1369)		0.5722	-0.5763 (-2.5761 to 1.4235)

Supplementary Table 8. GRADE assessment for primary outcomes

Certainty assessment							Summary of findings		
Outcome		Risk of bias	Inconsistency	Indirectness	Imprecision	Publication bias	Study (participant)	OR (95% CI)	GRADE of evidence
HHVs	PCL	Not serious	Serious[a]	Not serious	Not serious	Not serious	43 (6865/8352)	1.91 (1.54–2.36)	Very low
	CC	Not serious	Serious[a]	Not serious	Not serious	Serious[b]	55 (5802/11721)	2.81 (2.19–3.61)	Very low[c]
HSV-1	PCL	Not serious	Not serious	Not serious	Not serious	Not serious	7 (940/1513)	1.06 (0.81-1.39)	Low
	CC	Not serious	Serious[a]	Not serious	Not serious	Not serious	11(609/1250)	1.59(0.73-3.45)	Very low
HSV-2	PCL	Not serious	Serious[a]	Not serious	Not serious	Not serious	22 (3175/3927)	2.04 (1.46-2.84)	Low[c]
	CC	Not serious	Serious[a]	Not serious	Not serious	Serious[b]	38 (3991/8427)	3.13 (2.35-4.18)	Very low[c]
HCMV	PCL	Not serious	Not serious	Not serious	Not serious	Not serious	7 (1291/1202)	1.30 (0.83-2.02)	Low
	CC	Not serious	Not serious	Not serious	Not serious	Not serious	6 (611/932)	1.46 (0.80-2.64)	Low
EBV	PCL	Not serious	Not serious	Not serious	Not serious	Not serious	16 (770/1134)	3.55 (2.52-5.00)	Moderate[c]
	CC	Not serious	Serious[a]	Not serious	Not serious	Not serious	14 (470/719)	4.89 (2.18-10.96)	Low[c]

PCL: precancerous cervical lesions; CC: Cervical cancer; CI: Confidence interval; OR: Odds ratio

Explanations: In the GRADE approach, observation studies start as low-quality evidence[1]

a, I^2>50%; b. Peters test P<0.05; c, Upgrading, for large effects report if relative effect is >2 or >5.

Reference

1. Guyatt G, Oxman AD, Akl EA, Kunz R, Vist G, Brozek J, Norris S, Falck-Ytter Y, Glasziou P, DeBeer H, et al: **GRADE guidelines: 1. Introduction-GRADE evidence profiles and summary of findings tables.** *J Clin Epidemiol* 2011, **64:**383-394.
