# Supplementary material for: Association between human herpesvirus infection and cervical carcinoma: a systematic review and meta-analysis
Source: Virol J. 2023 Dec 4;20:288. doi: 10.1186/s12985-023-02234-5 (PMC10696706; doi:10.1186/s12985-023-02234-5)

Supplementary Figures

Supplementary Figure 1. Forest plot of the association between HHVs infections and cervical cancer (CC) or precancerous cervical lesions (PCL) risks in studies with matched controls, according to random effect model

Study	PCL or CC Events	PCL or CC Total	Controls Events	Controls Total	OR	95%−CI	Weight
**Population = PCL**							
Adam et al 1985	7	23	8	23	0.82	[0.24; 2.82]	0.7%
Ammatuna et al 2000	8	59	3	51	2.51	[0.63; 10.02]	0.6%
Aromseree et al 2015	67	170	11	82	4.20	[2.07; 8.50]	0.9%
Aurelian et al 1973	19	20	10	20	19.00	[2.12; 170.38]	0.4%
Bahena−Román et al 2020	66	268	26	205	2.25	[1.37; 3.70]	1.0%
Bahena−Román et al 2020	11	268	4	205	2.15	[0.67; 6.86]	0.7%
Becker et al 1996	207	255	262	332	1.15	[0.76; 1.74]	1.0%
Becker et al 1996	83	254	98	333	1.16	[0.82; 1.66]	1.0%
Bell et al 1978	17	17	15	15	1.13	[0.02; 60.37]	0.2%
Bell et al 1978	13	17	6	15	4.88	[1.06; 22.38]	0.6%
Biganzoli et al 2020	32	96	2	29	6.75	[1.51; 30.18]	0.6%
Biganzoli et al 2020	82	96	23	29	1.53	[0.53; 4.42]	0.8%
Broccolo et al 2008	77	103	48	66	1.11	[0.55; 2.24]	0.9%
Broccolo et al 2008	39	103	9	66	3.86	[1.72; 8.66]	0.9%
Broccolo et al 2008	5	103	7	66	0.43	[0.13; 1.42]	0.7%
Cameron et al 2018	23	64	40	266	3.17	[1.72; 5.84]	0.9%
Chan et al 1999	20	167	17	207	1.52	[0.77; 3.01]	0.9%
Chan et al 1999	13	167	19	207	0.84	[0.40; 1.75]	0.9%
Chavoshpour−Mamaghani et al 2021	22	124	26	179	1.27	[0.68; 2.36]	0.9%
Dale et al 1988	29	34	95	108	0.79	[0.26; 2.41]	0.7%
Daxnerova et al 2003	30	404	6	266	3.48	[1.43; 8.47]	0.8%
de Abreu et al 2016	0	71	8	614	0.50	[0.03; 8.74]	0.3%
de Abreu et al 2016	2	71	20	614	0.86	[0.20; 3.76]	0.6%
de Sanjosé et al 1994	231	249	225	242	0.97	[0.49; 1.93]	0.9%
de Sanjosé et al 1994	36	249	26	242	1.40	[0.82; 2.41]	1.0%
de Sanjosé et al 1994	269	276	268	270	0.29	[0.06; 1.39]	0.6%
de Sanjosé et al 1994	168	276	134	270	1.58	[1.12; 2.22]	1.0%
Feng et al 2021	5	6	20	159	34.75	[3.86; 312.87]	0.4%
Ferrera et al 1997	40	41	77	81	2.08	[0.22; 19.22]	0.4%
Ferrera et al 1997	42	42	79	83	4.81	[0.25; 91.50]	0.3%
Graham et al 1982	21	47	56	130	1.07	[0.55; 2.09]	0.9%
Gupta et al 1992	58	76	22	72	7.32	[3.53; 15.18]	0.9%
Janda et al 1973	6	18	10	18	0.40	[0.10; 1.54]	0.6%
Janda et al 1973	9	18	3	18	5.00	[1.07; 23.46]	0.6%
Khenchouche et al 2013	10	37	2	14	2.22	[0.42; 11.73]	0.5%
Kienka et al 2019	3	8	20	52	0.96	[0.21; 4.46]	0.6%
Kienka et al 2019	7	8	19	52	12.16	[1.39; 106.48]	0.4%
Landers et al 1993	4	75	0	25	3.21	[0.17; 61.73]	0.3%
Lehtinen et al 1989	9	15	18	30	1.00	[0.28; 3.54]	0.7%
Marinho−Dias et al 2013	3	43	3	18	0.38	[0.07; 2.07]	0.5%
Marinho−Dias et al 2013	2	43	0	18	2.23	[0.10; 48.76]	0.2%
McCormick et al 2015	20	32	8	15	1.46	[0.42; 5.05]	0.7%
McDonald et al 1974	18	39	16	39	1.23	[0.50; 3.02]	0.8%
McDonald et al 1974	12	39	15	39	0.71	[0.28; 1.82]	0.8%
Mendis et al 1981	14	22	11	28	2.70	[0.85; 8.57]	0.7%
Muñoz et al 1995	140	215	114	212	1.60	[1.09; 2.37]	1.0%
Muñoz et al 1995	75	234	58	213	1.26	[0.84; 1.90]	1.0%
Pérez et al 2006	8	153	5	79	0.82	[0.26; 2.58]	0.7%
Pérez et al 2006	39	153	12	79	1.91	[0.94; 3.90]	0.9%
Santos et al 2009	14	66	8	89	2.73	[1.07; 6.95]	0.8%
Sasagawa et al 2000	6	17	2	12	2.73	[0.44; 16.75]	0.5%
Sasagawa et al 2000	20	23	13	35	11.28	[2.80; 45.47]	0.6%
Seo et al 2005	3	20	0	20	8.20	[0.40; 169.90]	0.2%
Shimakage et al 2001	4	5	0	4	27.00	[0.85; 856.53]	0.2%
Shoji et al 1997	18	114	0	20	7.86	[0.46; 135.77]	0.3%
Silver et al 2011	6	9	36	246	11.67	[2.79; 48.77]	0.6%
Szkaradkiewicz et al 2004	11	22	8	26	2.25	[0.69; 7.32]	0.7%
Thiry et al 1977	52	88	11	51	5.25	[2.38; 11.59]	0.9%
Vonka et al 1984	33	221	61	472	1.18	[0.75; 1.87]	1.0%
Wilkie et al 1980	23	29	2	23	40.25	[7.31; 221.68]	0.5%
Zhao et al 2012	156	177	162	183	0.96	[0.51; 1.83]	0.9%
Zhao et al 2012	54	177	15	183	4.92	[2.65; 9.12]	0.9%
Zhao et al 2012	22	210	20	233	1.25	[0.66; 2.36]	0.9%
Zhao et al 2012	76	210	22	233	5.44	[3.23; 9.16]	1.0%
**Random effects model**		**6756**		**8326**	**1.95**	**[1.58; 2.41]**	**44.7%**
Heterogeneity: $I^2 = 66\%$, $\tau^2 = 0.4129$, $p < 0.01$							
**Population = CC**							
Adam et al 1974	32	50	84	186	2.16	[1.13; 4.12]	0.9%
Adelusi et al 1976	75	94	11	99	31.58	[14.13; 70.56]	0.9%
Arivananthan et al 1997	10	30	0	7	7.68	[0.40; 147.93]	0.3%
Arnheim et al 2011	123	603	417	2968	1.57	[1.25; 1.96]	1.1%
Aromseree et al 2015	14	40	11	82	3.48	[1.40; 8.62]	0.8%
Aurelian et al 1973	49	49	29	49	68.80	[4.01; 1180.11]	0.3%
Bahena−Román et al 2020	70	171	26	205	4.77	[2.86; 7.96]	1.0%
Bahena−Román et al 2020	19	171	4	205	6.28	[2.09; 18.84]	0.7%
Bell et al 1978	12	13	13	13	0.31	[0.01; 8.30]	0.2%
Bell et al 1978	10	13	6	13	3.89	[0.72; 21.06]	0.5%
Brock et al 1989	65	115	100	193	1.21	[0.76; 1.92]	1.0%
Brock et al 1989	82	115	76	193	3.83	[2.33; 6.29]	1.0%
Brock et al 1989	36	115	60	193	1.01	[0.61; 1.66]	1.0%
Chan et al 1999	0	30	17	207	0.18	[0.01; 3.05]	0.3%
Chan et al 1999	3	30	19	207	1.10	[0.30; 3.96]	0.7%
Chavoshpour−Mamaghani et al 2021	14	61	26	179	1.75	[0.85; 3.63]	0.9%
Choi et al 1977	11	57	17	166	2.10	[0.92; 4.79]	0.9%
Soumya et al 2023	74	109	22	26	0.38	[0.12; 1.20]	0.7%
Dale et al 1988	66	70	95	108	2.26	[0.71; 7.23]	0.7%
de Sanjosé et al 1994	149	150	147	149	2.03	[0.18; 22.60]	0.3%
de Sanjosé et al 1994	109	150	89	149	1.79	[1.10; 2.91]	1.0%
de Sanjosé et al 1994	217	223	226	238	1.92	[0.71; 5.21]	0.8%
de Sanjosé et al 1994	58	223	28	238	2.64	[1.61; 4.32]	1.0%
Farivar et al 2012	0	76	4	150	0.21	[0.01; 4.00]	0.3%
Ferrera et al 1997	47	48	89	93	2.11	[0.23; 19.44]	0.4%
Ferrera et al 1997	44	49	90	95	0.49	[0.13; 1.78]	0.7%
Graham et al 1982	83	134	56	130	2.15	[1.31; 3.52]	1.0%
Gupta et al 1992	31	51	10	47	5.74	[2.34; 14.06]	0.8%
Janda et al 1973	20	61	41	57	0.19	[0.09; 0.42]	0.9%
Janda et al 1973	30	61	11	57	4.05	[1.77; 9.26]	0.9%
Kahla et al 2012	13	44	5	81	6.37	[2.09; 19.40]	0.7%
Kalimo et al 1981	4	7	0	26	68.14	[2.99; 1554.27]	0.2%
Kawana et al 1976	14	14	13	14	3.22	[0.12; 86.09]	0.2%
Kawana et al 1976	8	14	6	14	1.78	[0.40; 7.94]	0.6%
Kessler et al 1974	132	350	84	350	1.92	[1.38; 2.66]	1.0%
Khenchouche et al 2013	40	58	2	14	13.33	[2.70; 65.84]	0.6%
Kumar et al 1980	29	35	18	32	3.76	[1.22; 11.55]	0.7%
Kumar et al 1980	34	35	22	32	15.45	[1.85; 129.33]	0.4%
Kumar et al 1980	33	35	20	32	9.90	[2.01; 48.87]	0.6%
Landers et al 1993	5	18	0	25	20.78	[1.07; 404.70]	0.3%
Lehtinen et al 1989	11	14	22	28	1.00	[0.21; 4.78]	0.6%
Lehtinen et al 1992	14	32	26	62	1.08	[0.46; 2.55]	0.8%
Lehtinen et al 1996	11	72	37	143	0.52	[0.25; 1.09]	0.9%
Lehtinen et al 2002	27	178	63	525	1.31	[0.81; 2.13]	1.0%
Marinho−Dias et al 2013	2	9	3	18	1.43	[0.19; 10.57]	0.4%
Marinho−Dias et al 2013	2	9	0	18	12.33	[0.53; 288.58]	0.2%
McCormick et al 2015	11	18	8	15	1.38	[0.34; 5.51]	0.6%
McDonald et al 1974	44	107	53	107	0.71	[0.41; 1.22]	1.0%
McDonald et al 1974	40	107	28	107	1.68	[0.94; 3.02]	1.0%
Mendis et al 1981	19	26	4	13	6.11	[1.42; 26.36]	0.6%
Mendis et al 1981	19	29	4	24	9.50	[2.54; 35.51]	0.7%
Mendis et al 1981	13	25	3	24	7.58	[1.79; 32.07]	0.6%
Mendis et al 1981	51	79	14	59	5.85	[2.75; 12.47]	0.9%
Muñoz et al 1995	91	121	95	141	1.47	[0.85; 2.53]	1.0%
Muñoz et al 1995	82	195	68	189	1.29	[0.86; 1.95]	1.0%
Nahmias et al 1974	37	57	12	82	10.79	[4.76; 24.48]	0.9%
Najem et al 1983	28	32	24	32	2.33	[0.62; 8.72]	0.7%
Najem et al 1983	22	32	12	32	3.67	[1.30; 10.32]	0.8%
Peng et al 1991	41	98	41	142	1.77	[1.03; 3.04]	1.0%
Pérez et al 2006	4	34	5	79	1.97	[0.50; 7.86]	0.6%
Pérez et al 2006	9	34	12	79	2.01	[0.76; 5.35]	0.8%
Santos et al 2009	9	14	8	89	18.23	[4.91; 67.72]	0.7%
Sasagawa et al 2000	7	14	2	12	5.00	[0.79; 31.63]	0.5%
Sasagawa et al 2000	23	31	13	35	4.87	[1.69; 14.00]	0.8%
Se Thoe et al 1993	5	8	0	15	48.71	[2.15; 1101.85]	0.2%
Seo et al 2005	1	56	0	20	1.11	[0.04; 28.31]	0.2%
Shimakage et al 2001	14	16	0	4	52.20	[2.10; 1300.33]	0.2%
Shoji et al 1997	13	60	0	20	11.65	[0.66; 205.48]	0.3%
Silver et al 2011	3	4	36	246	17.50	[1.77; 172.92]	0.4%
Skinner et al 1977	14	15	117	192	8.97	[1.16; 69.67]	0.4%
Skinner et al 1977	12	15	26	192	25.54	[6.75; 96.66]	0.7%
Thiry et al 1977	35	47	11	51	10.61	[4.16; 27.03]	0.8%
Vass−Sorensen et al 1984	11	44	5	30	1.67	[0.51; 5.41]	0.7%
Vestergaard et al 1972	131	135	100	115	4.91	[1.58; 15.26]	0.7%
Vestergaard et al 1972	115	135	54	115	6.50	[3.57; 11.83]	0.9%
Vonka et al 1984	2	21	61	472	0.71	[0.16; 3.12]	0.6%
Wilkie et al 1980	21	40	2	23	11.61	[2.40; 56.21]	0.6%
Zhang et al 1992	11	80	19	43	0.20	[0.08; 0.48]	0.8%
Zhao et al 2012	20	24	162	183	0.65	[0.20; 2.08]	0.7%
Zhao et al 2012	8	24	15	183	5.60	[2.06; 15.22]	0.8%
Zhao et al 2012	1	24	20	233	0.46	[0.06; 3.61]	0.4%
Zhao et al 2012	9	24	22	233	5.75	[2.26; 14.67]	0.8%
**Random effects model**		**5911**		**11747**	**2.74**	**[2.13; 3.53]**	**55.3%**
Heterogeneity: $I^2 = 79\%$, $\tau^2 = 0.9206$, $p < 0.01$							
**Random effects model**		**12667**		**20073**	**2.36**	**[1.99; 2.81]**	**100.0%**
Heterogeneity: $I^2 = 75\%$, $\tau^2 = 0.7204$, $p < 0.01$							
Test for subgroup differences: $\chi^2_1 = 4.12$, df = 1 ($p = 0.04$)							

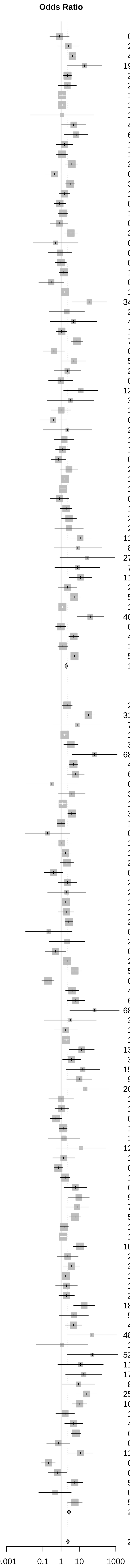

Supplementary Figure 2. Forest plot of the association between HSV-1 infection and cervical cancer (CC) or precancerous cervical lesions (PCL) risks in studies with matched controls, according to random effect model

Study	Method	Target	PCL or CC Events	PCL or CC Total	Controls Events	Controls Total	OR	95%−CI	Weight
**Population = PCL**									
Becker et al 1996	Immunological	antibodies	207	255	262	332	1.15	[0.76; 1.74]	8.2%
Bell et al 1978	Immunological	antibodies	17	17	15	15	1.13	[0.02; 60.37]	1.2%
de Abreu et al 2016	PCR−based	HSV−1 DNA	0	71	8	614	0.50	[0.03; 8.74]	2.0%
Janda et al 1973	Immunological	antibodies	6	18	10	18	0.40	[0.10; 1.54]	5.0%
McDonald et al 1974	Immunological	Antibodies	18	39	16	39	1.23	[0.50; 3.02]	6.6%
Pérez et al 2006	PCR−based	HSV−1 DNA	8	153	5	79	0.82	[0.26; 2.58]	5.6%
Zhao et al 2012	Immunological	Antibodies	156	177	162	183	0.96	[0.51; 1.83]	7.5%
Zhao et al 2012	PCR−based	HSV−1 DNA	22	210	20	233	1.25	[0.66; 2.36]	7.5%
**Random effects model**				940		1513	1.06	[0.81; 1.39]	43.4%
Heterogeneity: $I^2 = 0\%$, $\tau^2 = 0$, $p = 0.88$									
**Population = CC**									
Bell et al 1978	Immunological	antibodies	12	13	13	13	0.31	[0.01; 8.30]	1.6%
Brock et al 1989	Immunological	antibodies	82	115	76	193	3.83	[2.33; 6.29]	7.9%
Janda et al 1973	Immunological	antibodies	20	61	41	57	0.19	[0.09; 0.42]	7.0%
Kawana et al 1976	Immunological	Antibodies	14	14	13	14	3.22	[0.12; 86.09]	1.6%
Kumar et al 1980	Immunological	Antibodies	34	35	22	32	15.45	[1.85; 129.33]	3.1%
McDonald et al 1974	Immunological	Antibodies	44	107	53	107	0.71	[0.41; 1.22]	7.8%
Najem et al 1983	Immunological	Antibodies	28	32	24	32	2.33	[0.62; 8.72]	5.1%
Pérez et al 2006	PCR−based	HSV−1 DNA	4	34	5	79	1.97	[0.50; 7.86]	4.9%
Skinner et al 1977	Immunological	Antibodies	14	15	117	192	8.97	[1.16; 69.67]	3.2%
Vestergaard et al 1972	Immunological	Antibodies	131	135	100	115	4.91	[1.58; 15.26]	5.7%
Zhao et al 2012	PCR−based	HSV−1 DNA	1	24	20	233	0.46	[0.06; 3.61]	3.2%
Zhao et al 2012	Immunological	Antibodies	20	24	162	183	0.65	[0.20; 2.08]	5.6%
**Random effects model**				609		1250	1.59	[0.73; 3.45]	56.6%
Heterogeneity: $I^2 = 83\%$, $\tau^2 = 1.2631$, $p < 0.01$									
**Random effects model**				**1549**		**2763**	**1.23**	**[0.78; 1.95]**	**100.0%**

Heterogeneity: $I^2 = 72\%$, $\tau^2 = 0.6333$, $p < 0.01$
Test for subgroup differences: $\chi^2_1 = 0.91$, df = 1 ($p = 0.34$)

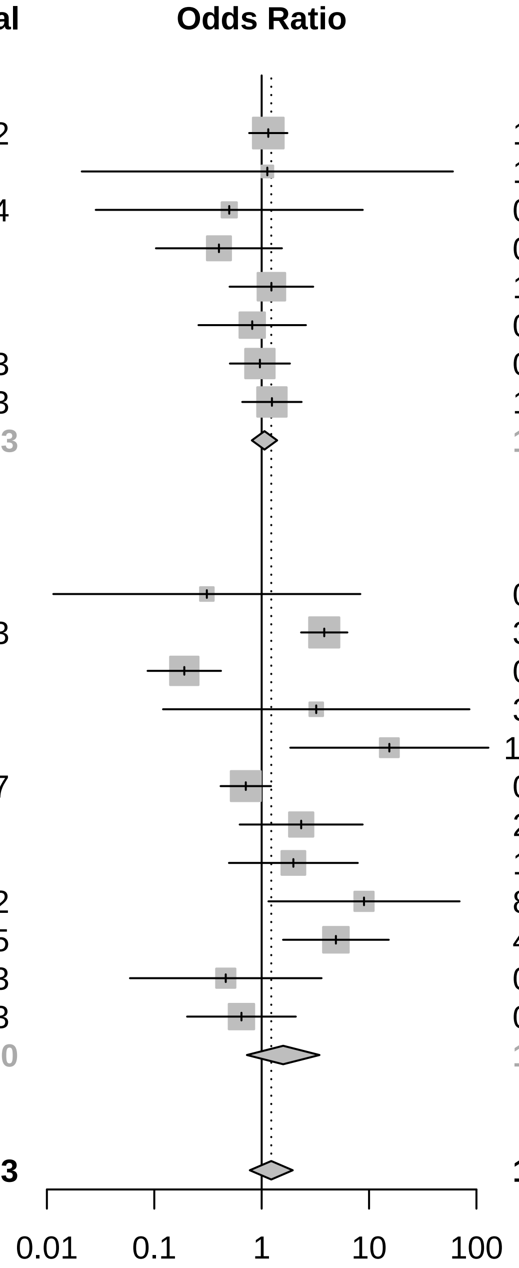

Supplementary Figure 3. Forest plot of the association between HCMV infection and cervical cancer (CC) or precancerous cervical lesions (PCL) risks in studies with matched controls, according to random effect model

Study	Method	Target	PCL or CC Events	PCL or CC Total	Controls Events	Controls Total	OR	95%−CI	Weight
**Population = PCL**									
Broccolo et al 2008	PCR−based	HCMV DNA	77	103	48	66	1.11	[0.55; 2.24]	12.7%
Chan et al 1999	PCR−based	mtrII	20	167	17	207	1.52	[0.77; 3.01]	13.1%
Daxnerova et al 2003	PCR−based	HCMV DNA	30	404	6	266	3.48	[1.43; 8.47]	9.0%
de Sanjosé et al 1994	Immunological	antibodies	231	249	225	242	0.97	[0.49; 1.93]	13.0%
de Sanjosé et al 1994	Immunological	antibodies	269	276	268	270	0.29	[0.06; 1.39]	3.4%
Ferrera et al 1997	Immunological	Antibodies	40	41	77	81	2.08	[0.22; 19.22]	1.8%
Kienka et al 2019	PCR−based	HCMV DNA	3	8	20	52	0.96	[0.21; 4.46]	3.6%
Marinho−Dias et al 2013	PCR−based	US17	2	43	0	18	2.23	[0.10; 48.76]	1.0%
**Random effects model**				1291		1202	1.30	[0.83; 2.02]	57.6%
Heterogeneity: $I^2 = 28\%$, $\tau^2 = 0.1134$, $p = 0.20$									
**Population = CC**									
Brock et al 1989	Immunological	antibodies	65	115	100	193	1.21	[0.76; 1.92]	20.1%
Chan et al 1999	PCR−based	mtrII	0	30	17	207	0.18	[0.01; 3.05]	1.1%
de Sanjosé et al 1994	Immunological	antibodies	149	150	147	149	2.03	[0.18; 22.60]	1.6%
de Sanjosé et al 1994	Immunological	antibodies	217	223	226	238	1.92	[0.71; 5.21]	7.6%
Ferrera et al 1997	Immunological	Antibodies	44	49	90	95	0.49	[0.13; 1.78]	4.9%
Kumar et al 1980	Immunological	Antibodies	29	35	18	32	3.76	[1.22; 11.55]	6.2%
Marinho−Dias et al 2013	PCR−based	US17	2	9	0	18	12.33	[0.53;288.58]	0.9%
**Random effects model**				611		932	1.46	[0.80; 2.64]	42.4%
Heterogeneity: $I^2 = 41\%$, $\tau^2 = 0.2053$, $p = 0.12$									
**Random effects model**				**1902**		**2134**	**1.35**	**[1.00; 1.84]**	**100.0%**
Heterogeneity: $I^2 = 30\%$, $\tau^2 = 0.0664$, $p = 0.13$									
Test for subgroup differences: $\chi^2_1 = 0.09$, df = 1 ($p = 0.76$)									

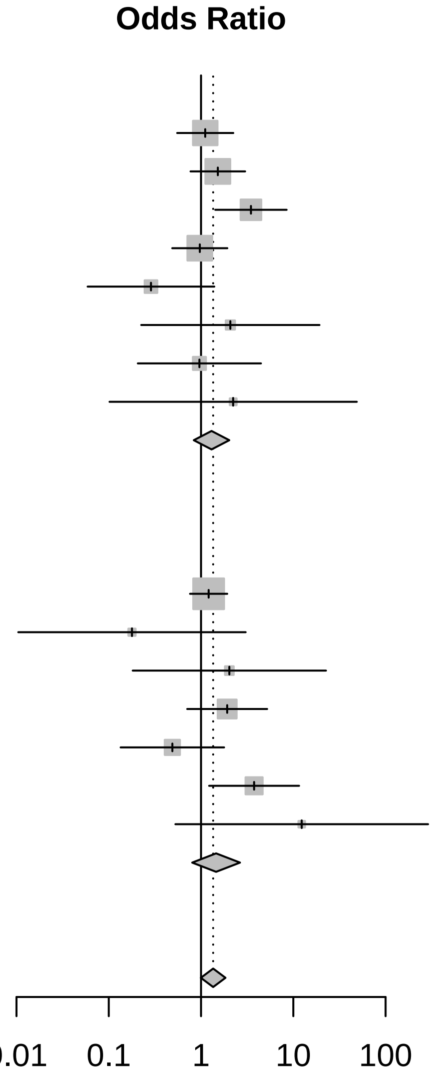

Supplementary Figure 4. Forest plots showing the adjusted odds ratios for risk of cervical cancer (CC) or precancerous cervical lesions (PCL) incidences and exposure to HSV-2 infection

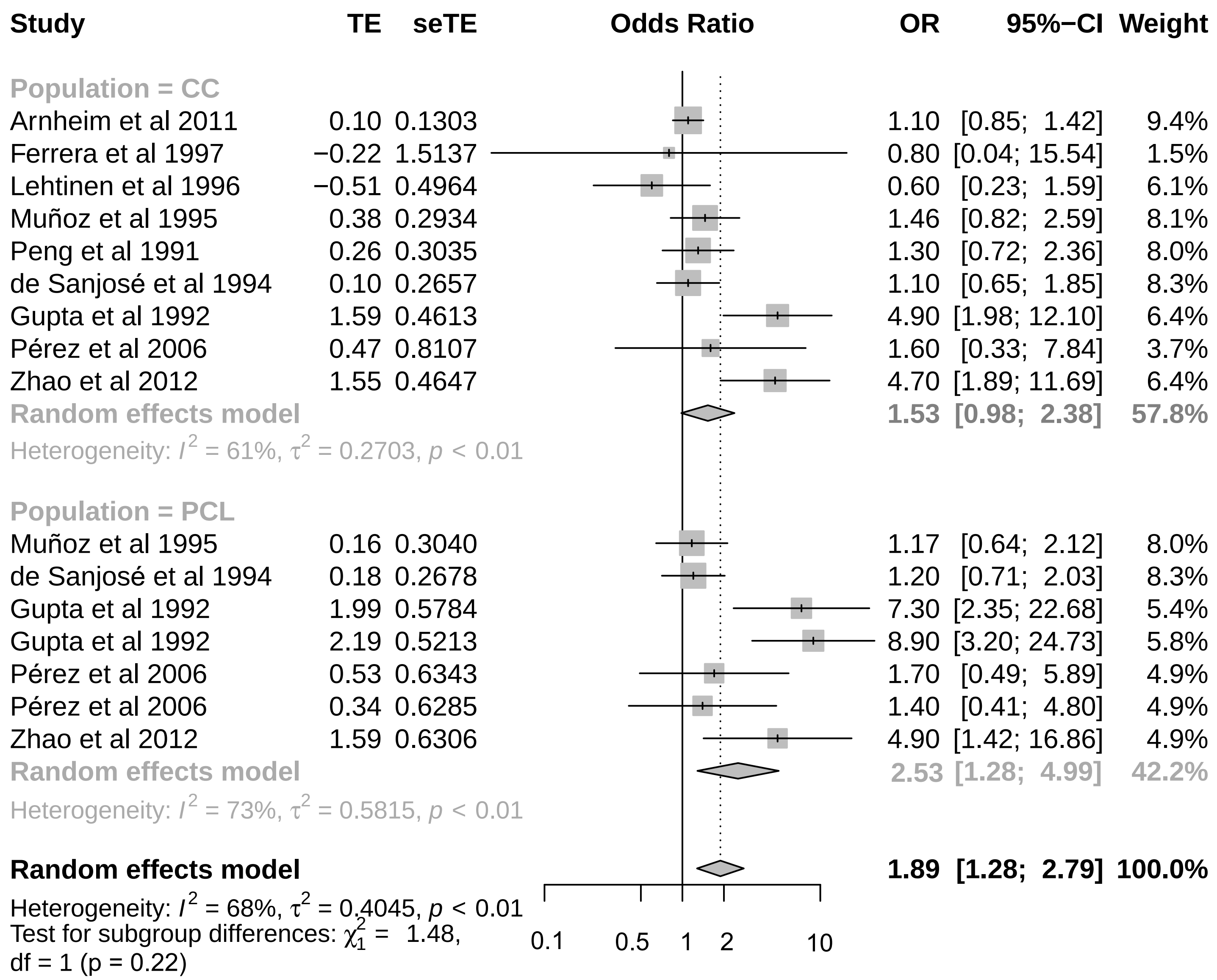

Supplementary Figure 5. HSV-2 and cervical cancer (CC) or precancerous cervical lesions (PCL): subgroup meta-analyses forest plots according to different stages of disease (A), viral detection methods (B), specimen types (C), and different HDI regions (D)

A.

Study	PCL or CC Events	PCL or CC Total	Controls Events	Controls Total	OR	95%−CI	Weight
**Population = CIN 1**							
Bahena−Román 2020	8	228	4	205	1.83	[0.54; 6.16]	1.4%
Bahena−Román 2020	55	231	26	205	2.15	[1.29; 3.59]	2.0%
Becker 1996	12	57	98	333	0.64	[0.32; 1.26]	1.9%
Ferrera 1997	9	9	18	18	0.51	[0.01; 27.96]	0.3%
Gupta 1992	25	35	22	72	5.68	[2.34; 13.81]	1.7%
Pérez 2006	22	79	12	79	2.15	[0.98; 4.73]	1.8%
**Random effects model**		639		912	1.87	[0.95; 3.70]	9.0%
Heterogeneity: $I^2 = 70\%$, $\tau^2 = 0.4475$, $p < 0.01$							
**Population = CIN 2/3**							
Bahena−Román 2020	11	37	26	205	2.91	[1.29; 6.59]	1.7%
Bahena−Román 2020	3	40	4	205	4.07	[0.88; 18.96]	1.1%
Becker 1996	71	197	98	333	1.35	[0.93; 1.96]	2.1%
de Abreu 2016	2	71	20	614	0.86	[0.20; 3.76]	1.2%
de Sanjosé 1994	36	249	26	242	1.40	[0.82; 2.41]	2.0%
de Sanjosé 1994	168	276	134	270	1.58	[1.12; 2.22]	2.1%
Ferrera 1997	33	33	61	65	4.90	[0.26; 93.84]	0.5%
Gupta 1992	33	41	22	72	9.37	[3.73; 23.55]	1.6%
Lehtinen 1989	9	15	18	30	1.00	[0.28; 3.54]	1.3%
Muñoz 1995	75	234	58	213	1.26	[0.84; 1.90]	2.1%
Muñoz 1995	140	215	114	212	1.60	[1.09; 2.37]	2.1%
Pérez 2006	17	74	12	79	1.67	[0.73; 3.78]	1.7%
Vonka 1984	33	221	61	472	1.18	[0.75; 1.87]	2.1%
**Random effects model**		1703		3012	1.64	[1.29; 2.08]	21.6%
Heterogeneity: $I^2 = 48\%$, $\tau^2 = 0.0726$, $p = 0.03$							
**Population = CC**							
Adam 1974	32	50	84	186	2.16	[1.13; 4.12]	1.9%
Adelusi 1976	75	94	11	99	31.58	[14.13; 70.56]	1.8%
Arnheim 2011	123	603	417	2968	1.57	[1.25; 1.96]	2.2%
Aurelian 1973	49	49	29	49	68.80	[4.01; 1180.11]	0.5%
Bahena−Román 2020	19	171	4	205	6.28	[2.09; 18.84]	1.5%
Bahena−Román 2020	70	171	26	205	4.77	[2.86; 7.96]	2.0%
Bell 1978	10	13	6	13	3.89	[0.72; 21.06]	1.0%
Brock 1989	36	115	60	193	1.01	[0.61; 1.66]	2.0%
Choi 1977	11	57	17	166	2.10	[0.92; 4.79]	1.7%
Soumya 2023	74	109	22	26	0.38	[0.12; 1.20]	1.4%
Dale 1988	66	70	95	108	2.26	[0.71; 7.23]	1.4%
de Sanjosé 1994	58	223	28	238	2.64	[1.61; 4.32]	2.0%
de Sanjosé 1994	109	150	89	149	1.79	[1.10; 2.91]	2.0%
Farivar 2012	0	76	4	150	0.21	[0.01; 4.00]	0.5%
Ferrera 1997	47	48	89	93	2.11	[0.23; 19.44]	0.7%
Graham 1982	83	134	56	130	2.15	[1.31; 3.52]	2.0%
Gupta 1992	31	51	10	47	5.74	[2.34; 14.06]	1.7%
Janda 1973	30	61	11	57	4.05	[1.77; 9.26]	1.7%
Kalimo 1981	4	7	0	26	68.14	[2.99; 1554.27]	0.4%
Kawana 1976	8	14	6	14	1.78	[0.40; 7.94]	1.1%
Kessler 1974	132	350	84	350	1.92	[1.38; 2.66]	2.1%
Kumar 1980	33	35	20	32	9.90	[2.01; 48.87]	1.1%
Lehtinen 1989	11	14	22	28	1.00	[0.21; 4.78]	1.1%
Lehtinen 1992	14	32	26	62	1.08	[0.46; 2.55]	1.7%
Lehtinen 1996	11	72	37	143	0.52	[0.25; 1.09]	1.8%
Lehtinen 2002	27	178	63	525	1.31	[0.81; 2.13]	2.0%
McDonald 1974	40	107	28	107	1.68	[0.94; 3.02]	2.0%
Mendis 1981	13	25	3	24	7.58	[1.79; 32.07]	1.2%
Mendis 1981	19	29	4	24	9.50	[2.54; 35.51]	1.3%
Mendis 1981	19	26	4	13	6.11	[1.42; 26.36]	1.2%
Mendis 1981	51	79	14	59	5.85	[2.75; 12.47]	1.8%
Muñoz 1995	82	195	68	189	1.29	[0.86; 1.95]	2.1%
Muñoz 1995	91	121	95	141	1.47	[0.85; 2.53]	2.0%
Nahmias 1974	37	57	12	82	10.79	[4.76; 24.48]	1.7%
Najem 1983	22	32	12	32	3.67	[1.30; 10.32]	1.5%
Peng 1991	41	98	41	142	1.77	[1.03; 3.04]	2.0%
Pérez 2006	9	34	12	79	2.01	[0.76; 5.35]	1.6%
Skinner 1977	12	15	26	192	25.54	[6.75; 96.66]	1.3%
Thiry 1977	35	47	11	51	10.61	[4.16; 27.03]	1.6%
Vass−Sorensen 1984	11	44	5	30	1.67	[0.51; 5.41]	1.4%
Vestergaard 1972	115	135	54	115	6.50	[3.57; 11.83]	1.9%
Vonka 1984	2	21	61	472	0.71	[0.16; 3.12]	1.1%
Wilkie 1980	21	40	2	23	11.61	[2.40; 56.21]	1.1%
Zhao 2012	8	24	15	183	5.60	[2.06; 15.22]	1.6%
Zhao 2012	9	24	22	233	5.75	[2.26; 14.67]	1.6%
**Random effects model**		4100		8453	3.01	[2.24; 4.04]	69.4%
Heterogeneity: $I^2 = 80\%$, $\tau^2 = 0.7345$, $p < 0.01$							
**Random effects model**		**6442**		**12377**	**2.56**	**[2.04; 3.21]**	**100.0%**
Heterogeneity: $I^2 = 77\%$, $\tau^2 = 0.6014$, $p < 0.01$							
Test for subgroup differences: $\chi^2_2 = 10.03$, df = 2 ($p < 0.01$)							

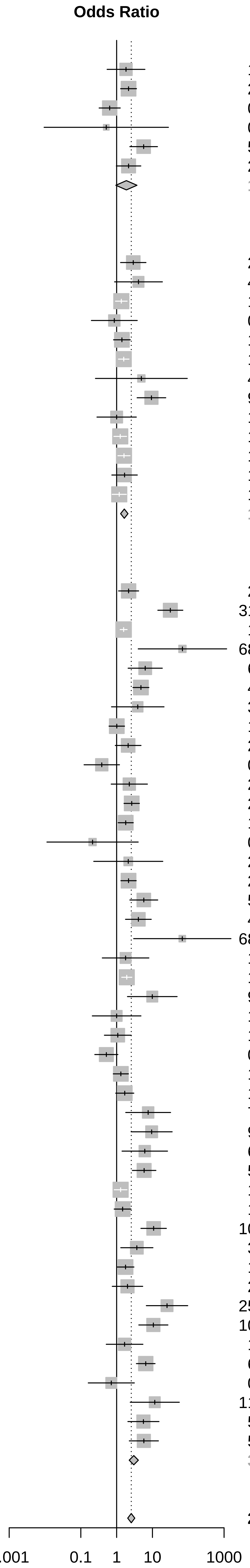

B.

Study	PCL or CC Events	PCL or CC Total	Controls Events	Controls Total	OR	95%−CI	Weight
**Method = Immunological**							
Adam 1985	7	23	8	23	0.82	[0.24; 2.82]	1.1%
Adam 1974	32	50	84	186	2.16	[1.13; 4.12]	1.6%
Adelusi 1976	75	94	11	99	31.58	[14.13; 70.56]	1.5%
Arnheim 2011	123	603	417	2968	1.57	[1.25; 1.96]	1.9%
Aurelian 1973	19	20	10	20	19.00	[2.12; 170.38]	0.6%
Aurelian 1973	49	49	29	49	68.80	[4.01; 1180.11]	0.4%
Bahena−Román 2020	70	171	26	205	4.77	[2.86; 7.96]	1.7%
Bahena−Román 2020	55	231	26	205	2.15	[1.29; 3.59]	1.7%
Bahena−Román 2020	11	37	26	205	2.91	[1.29; 6.59]	1.5%
Becker 1996	12	57	98	333	0.64	[0.32; 1.26]	1.6%
Becker 1996	23	73	98	333	1.10	[0.64; 1.91]	1.7%
Becker 1996	48	124	98	333	1.51	[0.98; 2.33]	1.8%
Bell 1978	10	13	6	13	3.89	[0.72; 21.06]	0.8%
Bell 1978	13	17	6	15	4.88	[1.06; 22.38]	0.9%
Brock 1989	36	115	60	193	1.01	[0.61; 1.66]	1.7%
Choi 1977	11	57	17	166	2.10	[0.92; 4.79]	1.5%
Dale 1988	66	70	95	108	2.26	[0.71; 7.23]	1.2%
Dale 1988	29	34	95	108	0.79	[0.26; 2.41]	1.2%
de Sanjosé 1994	109	150	89	149	1.79	[1.10; 2.91]	1.7%
de Sanjosé 1994	58	223	28	238	2.64	[1.61; 4.32]	1.7%
de Sanjosé 1994	36	249	26	242	1.40	[0.82; 2.41]	1.7%
de Sanjosé 1994	168	276	134	270	1.58	[1.12; 2.22]	1.8%
Ferrera 1997	8	8	15	15	0.55	[0.01; 30.19]	0.2%
Ferrera 1997	9	9	18	18	0.51	[0.01; 27.96]	0.2%
Ferrera 1997	25	25	46	50	4.94	[0.26; 95.38]	0.4%
Ferrera 1997	47	48	89	93	2.11	[0.23; 19.44]	0.6%
Graham 1982	83	134	56	130	2.15	[1.31; 3.52]	1.7%
Graham 1982	21	47	56	130	1.07	[0.55; 2.09]	1.6%
Gupta 1992	31	51	10	47	5.74	[2.34; 14.06]	1.4%
Gupta 1992	33	41	22	72	9.37	[3.73; 23.55]	1.4%
Gupta 1992	25	35	22	72	5.68	[2.34; 13.81]	1.4%
Janda 1973	9	18	3	18	5.00	[1.07; 23.46]	0.9%
Janda 1973	30	61	11	57	4.05	[1.77; 9.26]	1.5%
Kawana 1976	8	14	6	14	1.78	[0.40; 7.94]	0.9%
Kessler 1974	132	350	84	350	1.92	[1.38; 2.66]	1.8%
Kumar 1980	33	35	20	32	9.90	[2.01; 48.87]	0.9%
Lehtinen 1989	11	14	22	28	1.00	[0.21; 4.78]	0.9%
Lehtinen 1989	9	15	18	30	1.00	[0.28; 3.54]	1.1%
Lehtinen 1992	14	32	26	62	1.08	[0.46; 2.55]	1.4%
Lehtinen 1996	11	72	37	143	0.52	[0.25; 1.09]	1.5%
Lehtinen 2002	27	178	63	525	1.31	[0.81; 2.13]	1.7%
McDonald 1974	12	39	15	39	0.71	[0.28; 1.82]	1.4%
McDonald 1974	40	107	28	107	1.68	[0.94; 3.02]	1.7%
Mendis 1981	19	26	4	13	6.11	[1.42; 26.36]	1.0%
Mendis 1981	19	29	4	24	9.50	[2.54; 35.51]	1.1%
Mendis 1981	13	25	3	24	7.58	[1.79; 32.07]	1.0%
Mendis 1981	14	22	11	28	2.70	[0.85; 8.57]	1.2%
Mendis 1981	51	79	14	59	5.85	[2.75; 12.47]	1.5%
Muñoz 1995	91	121	95	141	1.47	[0.85; 2.53]	1.7%
Muñoz 1995	82	195	68	189	1.29	[0.86; 1.95]	1.8%
Muñoz 1995	140	215	114	212	1.60	[1.09; 2.37]	1.8%
Muñoz 1995	75	234	58	213	1.26	[0.84; 1.90]	1.8%
Nahmias 1974	37	57	12	82	10.79	[4.76; 24.48]	1.5%
Najem 1983	22	32	12	32	3.67	[1.30; 10.32]	1.3%
Peng 1991	41	98	41	142	1.77	[1.03; 3.04]	1.7%
Skinner 1977	12	15	26	192	25.54	[6.75; 96.66]	1.1%
Soumya 2023	74	109	22	26	0.38	[0.12; 1.20]	1.2%
Thiry 1977	35	47	11	51	10.61	[4.16; 27.03]	1.4%
Thiry 1977	52	88	11	51	5.25	[2.38; 11.59]	1.5%
Vass−Sorensen 1984	11	44	5	30	1.67	[0.51; 5.41]	1.2%
Vestergaard 1972	115	135	54	115	6.50	[3.57; 11.83]	1.6%
Vonka 1984	14	76	30	205	1.32	[0.66; 2.65]	1.6%
Vonka 1984	2	21	30	205	0.61	[0.14; 2.77]	0.9%
Vonka 1984	19	145	31	267	1.15	[0.62; 2.11]	1.6%
Zhao 2012	8	24	15	183	5.60	[2.06; 15.22]	1.3%
Zhao 2012	54	177	15	183	4.92	[2.65; 9.12]	1.6%
**Random effects model**		6083		11160	2.41	[1.92; 3.02]	88.0%
Heterogeneity: $I^2 = 77\%$, $\tau^2 = 0.6173$, $p < 0.01$							
**Method = PCR−based**							
Bahena−Román 2020	19	171	4	205	6.28	[2.09; 18.84]	1.2%
Bahena−Román 2020	8	228	4	205	1.83	[0.54; 6.16]	1.1%
Bahena−Román 2020	3	40	4	205	4.07	[0.88; 18.96]	0.9%
de Abreu 2016	2	71	20	614	0.86	[0.20; 3.76]	1.0%
Farivar 2012	0	76	4	150	0.21	[0.01; 4.00]	0.4%
Pérez 2006	9	34	12	79	2.01	[0.76; 5.35]	1.3%
Pérez 2006	22	79	12	79	2.15	[0.98; 4.73]	1.5%
Pérez 2006	17	74	12	79	1.67	[0.73; 3.78]	1.5%
Zhao 2012	9	24	22	233	5.75	[2.26; 14.67]	1.4%
Zhao 2012	76	210	22	233	5.44	[3.23; 9.16]	1.7%
**Random effects model**		1007		2082	2.79	[1.79; 4.34]	12.0%
Heterogeneity: $I^2 = 51\%$, $\tau^2 = 0.2152$, $p = 0.03$							
**Random effects model**		**7090**		**13242**	**2.43**	**[1.98; 2.98]**	**100.0%**

Heterogeneity: $I^2 = 76\%$, $\tau^2 = 0.5667$, $p < 0.01$
Test for subgroup differences: $\chi^2_1 = 0.33$, df = 1 ($p = 0.56$)

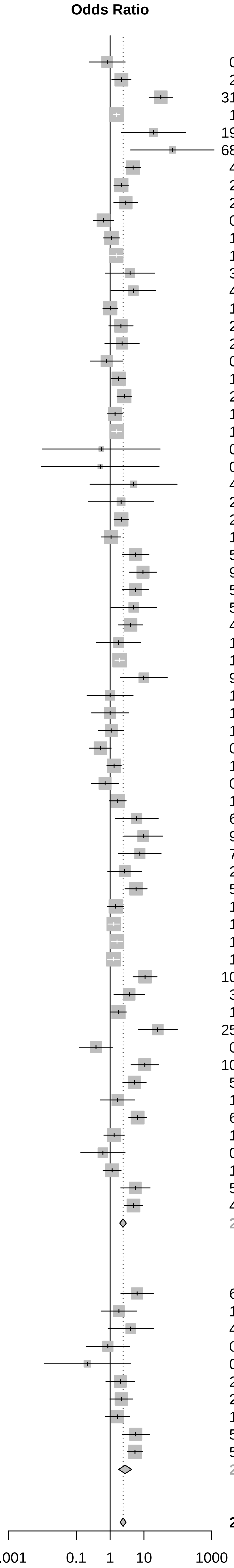

C.

Study	PCL or CC Events	PCL or CC Total	Controls Events	Controls Total	Odds Ratio	OR	95%−CI	Weight
**Specimen = Serum**								
Adam et al 1974	32	50	84	186		2.16	[1.13; 4.12]	1.8%
Adam et al 1985	7	23	8	23		0.82	[0.24; 2.82]	1.3%
Adelusi et al 1976	75	94	11	99		31.58	[14.13; 70.56]	1.6%
Arnheim et al 2011	123	603	417	2968		1.57	[1.25; 1.96]	2.1%
Aurelian et al 1973	19	20	10	20		19.00	[2.12; 170.38]	0.7%
Aurelian et al 1973	49	49	29	49		68.80	[4.01; 1180.11]	0.5%
Bahena−Román et al 2020	66	268	26	205		2.25	[1.37; 3.70]	1.9%
Bahena−Román et al 2020	70	171	26	205		4.77	[2.86; 7.96]	1.9%
Becker et al 1996	83	254	98	333		1.16	[0.82; 1.66]	2.0%
Bell et al 1978	13	17	6	15		4.88	[1.06; 22.38]	1.0%
Bell et al 1978	10	13	6	13		3.89	[0.72; 21.06]	0.9%
Brock et al 1989	36	115	60	193		1.01	[0.61; 1.66]	1.9%
Choi et al 1977	11	57	17	166		2.10	[0.92; 4.79]	1.6%
Dale et al 1988	29	34	95	108		0.79	[0.26; 2.41]	1.4%
Dale et al 1988	66	70	95	108		2.26	[0.71; 7.23]	1.3%
de Sanjosé et al 1994	36	249	26	242		1.40	[0.82; 2.41]	1.9%
de Sanjosé et al 1994	168	276	134	270		1.58	[1.12; 2.22]	2.0%
de Sanjosé et al 1994	109	150	89	149		1.79	[1.10; 2.91]	1.9%
de Sanjosé et al 1994	58	223	28	238		2.64	[1.61; 4.32]	1.9%
Ferrera et al 1997	42	42	79	83		4.81	[0.25; 91.50]	0.4%
Ferrera et al 1997	47	48	89	93		2.11	[0.23; 19.44]	0.7%
Graham et al 1982	21	47	56	130		1.07	[0.55; 2.09]	1.8%
Graham et al 1982	83	134	56	130		2.15	[1.31; 3.52]	1.9%
Gupta et al 1992	58	76	22	72		7.32	[3.53; 15.18]	1.7%
Gupta et al 1992	31	51	10	47		5.74	[2.34; 14.06]	1.6%
Janda et al 1973	9	18	3	18		5.00	[1.07; 23.46]	1.0%
Janda et al 1973	30	61	11	57		4.05	[1.77; 9.26]	1.6%
Kawana et al 1976	8	14	6	14		1.78	[0.40; 7.94]	1.1%
Kessler et al 1974	132	350	84	350		1.92	[1.38; 2.66]	2.0%
Kumar et al 1980	33	35	20	32		9.90	[2.01; 48.87]	1.0%
Lehtinen et al 1989	9	15	18	30		1.00	[0.28; 3.54]	1.2%
Lehtinen et al 1989	11	14	22	28		1.00	[0.21; 4.78]	1.0%
Lehtinen et al 1992	14	32	26	62		1.08	[0.46; 2.55]	1.6%
Lehtinen et al 1996	11	72	37	143		0.52	[0.25; 1.09]	1.7%
Lehtinen et al 2002	27	178	63	525		1.31	[0.81; 2.13]	1.9%
McDonald et al 1974	12	39	15	39		0.71	[0.28; 1.82]	1.5%
McDonald et al 1974	40	107	28	107		1.68	[0.94; 3.02]	1.8%
Mendis et al 1981	14	22	11	28		2.70	[0.85; 8.57]	1.3%
Mendis et al 1981	19	26	4	13		6.11	[1.42; 26.36]	1.1%
Mendis et al 1981	19	29	4	24		9.50	[2.54; 35.51]	1.2%
Mendis et al 1981	13	25	3	24		7.58	[1.79; 32.07]	1.1%
Mendis et al 1981	51	79	14	59		5.85	[2.75; 12.47]	1.7%
Muñoz et al 1995	140	215	114	212		1.60	[1.09; 2.37]	2.0%
Muñoz et al 1995	75	234	58	213		1.26	[0.84; 1.90]	2.0%
Muñoz et al 1995	91	121	95	141		1.47	[0.85; 2.53]	1.9%
Muñoz et al 1995	82	195	68	189		1.29	[0.86; 1.95]	2.0%
Nahmias et al 1974	37	57	12	82		10.79	[4.76; 24.48]	1.6%
Najem et al 1983	22	32	12	32		3.67	[1.30; 10.32]	1.4%
Peng et al 1991	41	98	41	142		1.77	[1.03; 3.04]	1.9%
Skinner et al 1977	12	15	26	192		25.54	[6.75; 96.66]	1.2%
Soumya et al 2023	74	109	22	26		0.38	[0.12; 1.20]	1.3%
Thiry et al 1977	52	88	11	51		5.25	[2.38; 11.59]	1.7%
Thiry et al 1977	35	47	11	51		10.61	[4.16; 27.03]	1.5%
Vass−Sorensen et al 1984	11	44	5	30		1.67	[0.51; 5.41]	1.3%
Vestergaard et al 1972	115	135	54	115		6.50	[3.57; 11.83]	1.8%
Vonka et al 1984	33	221	61	472		1.18	[0.75; 1.87]	1.9%
Vonka et al 1984	2	21	61	472		0.71	[0.16; 3.12]	1.1%
Zhao et al 2012	54	177	15	183		4.92	[2.65; 9.12]	1.8%
Zhao et al 2012	8	24	15	183		5.60	[2.06; 15.22]	1.5%
**Random effects model**		**6083**		**10484**		**2.50**	**[1.98; 3.17]**	**89.2%**
Heterogeneity: $I^2 = 79\%$, $\tau^2 = 0.6261$, $p < 0.01$								
**Specimen = Brush/swab**								
Bahena−Román et al 2020	11	268	4	205		2.15	[0.67; 6.86]	1.3%
Bahena−Román et al 2020	19	171	4	205		6.28	[2.09; 18.84]	1.4%
de Abreu et al 2016	2	71	20	614		0.86	[0.20; 3.76]	1.1%
Kalimo et al 1981	4	7	0	26		68.14	[2.99; 1554.27]	0.4%
Pérez et al 2006	39	153	12	79		1.91	[0.94; 3.90]	1.7%
Pérez et al 2006	9	34	12	79		2.01	[0.76; 5.35]	1.5%
Zhao et al 2012	76	210	22	233		5.44	[3.23; 9.16]	1.9%
Zhao et al 2012	9	24	22	233		5.75	[2.26; 14.67]	1.5%
**Random effects model**		**938**		**1674**		**3.34**	**[2.02; 5.53]**	**10.8%**
Heterogeneity: $I^2 = 58\%$, $\tau^2 = 0.2426$, $p = 0.02$								
**Random effects model**		**7021**		**12158**		**2.58**	**[2.07; 3.21]**	**100.0%**
Heterogeneity: $I^2 = 78\%$, $\tau^2 = 0.5923$, $p < 0.01$								
Test for subgroup differences: $\chi^2_1 = 1.02$, df = 1 ($p = 0.31$)								

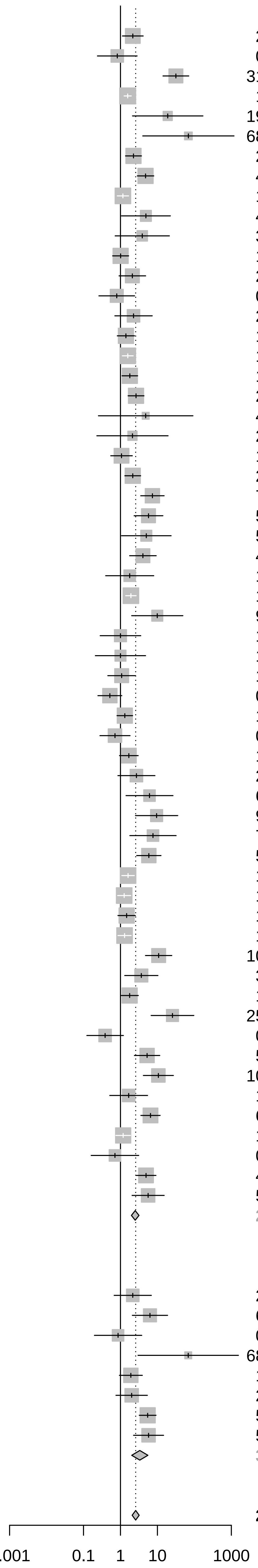

D.

Study	PCL or CC Events	PCL or CC Total	Controls Events	Controls Total	OR	95%-CI	Weight
**HDI = Low HDI**							
Adelusi 1976	75	94	11	99	31.58	[14.13; 70.56]	1.4%
Mendis 1981	19	26	4	13	6.11	[1.42; 26.36]	1.0%
**Random effects model**		120		112	15.63	[3.18; 76.90]	2.4%
Heterogeneity: I^2 = 73%, τ^2 = 0.9874, p = 0.05							
**HDI = Medium HDI**							
Ferrera 1997	8	8	15	15	0.55	[0.01; 30.19]	0.2%
Ferrera 1997	9	9	18	18	0.51	[0.01; 27.96]	0.2%
Ferrera 1997	25	25	46	50	4.94	[0.26; 95.38]	0.4%
Ferrera 1997	47	48	89	93	2.11	[0.23; 19.44]	0.6%
Gupta 1992	31	51	10	47	5.74	[2.34; 14.06]	1.4%
Gupta 1992	33	41	22	72	9.37	[3.73; 23.55]	1.4%
Gupta 1992	25	35	22	72	5.68	[2.34; 13.81]	1.4%
Soumya 2023	74	109	22	26	0.38	[0.12; 1.20]	1.2%
**Random effects model**		326		393	2.82	[1.05; 7.54]	6.8%
Heterogeneity: I^2 = 70%, τ^2 = 1.1475, p < 0.01							
**HDI = High HDI**							
Bahena−Román 2020	19	171	4	205	6.28	[2.09; 18.84]	1.2%
Bahena−Román 2020	8	228	4	205	1.83	[0.54; 6.16]	1.1%
Bahena−Román 2020	3	40	4	205	4.07	[0.88; 18.96]	0.9%
Bahena−Román 2020	70	171	26	205	4.77	[2.86; 7.96]	1.7%
Bahena−Román 2020	55	231	26	205	2.15	[1.29; 3.59]	1.7%
Bahena−Román 2020	11	37	26	205	2.91	[1.29; 6.59]	1.4%
de Sanjosé 1994	109	150	89	149	1.79	[1.10; 2.91]	1.7%
de Sanjosé 1994	168	276	134	270	1.58	[1.12; 2.22]	1.7%
Farivar 2012	0	76	4	150	0.21	[0.01; 4.00]	0.4%
Mendis 1981	19	29	4	24	9.50	[2.54; 35.51]	1.1%
Muñoz 1995	91	121	95	141	1.47	[0.85; 2.53]	1.6%
Muñoz 1995	140	215	114	212	1.60	[1.09; 2.37]	1.7%
Peng 1991	41	98	41	142	1.77	[1.03; 3.04]	1.6%
Zhao 2012	8	24	15	183	5.60	[2.06; 15.22]	1.3%
Zhao 2012	54	177	15	183	4.92	[2.65; 9.12]	1.6%
Zhao 2012	9	24	22	233	5.75	[2.26; 14.67]	1.3%
Zhao 2012	76	210	22	233	5.44	[3.23; 9.16]	1.6%
**Random effects model**		2278		3150	2.84	[2.10; 3.82]	23.7%
Heterogeneity: I^2 = 70%, τ^2 = 0.2373, p < 0.01							
**HDI = Very high HDI**							
Adam 1985	7	23	8	23	0.82	[0.24; 2.82]	1.1%
Adam 1974	32	50	84	186	2.16	[1.13; 4.12]	1.6%
Arnheim 2011	123	603	417	2968	1.57	[1.25; 1.96]	1.8%
Aurelian 1973	19	20	10	20	19.00	[2.12; 170.38]	0.6%
Aurelian 1973	49	49	29	49	68.80	[4.01; 1180.11]	0.4%
Becker 1996	12	57	98	333	0.64	[0.32; 1.26]	1.5%
Becker 1996	23	73	98	333	1.10	[0.64; 1.91]	1.6%
Becker 1996	48	124	98	333	1.51	[0.98; 2.33]	1.7%
Bell 1978	10	13	6	13	3.89	[0.72; 21.06]	0.8%
Bell 1978	13	17	6	15	4.88	[1.06; 22.38]	0.9%
Brock 1989	36	115	60	193	1.01	[0.61; 1.66]	1.7%
Choi 1977	11	57	17	166	2.10	[0.92; 4.79]	1.4%
Dale 1988	66	70	95	108	2.26	[0.71; 7.23]	1.2%
Dale 1988	29	34	95	108	0.79	[0.26; 2.41]	1.2%
de Abreu 2016	2	71	20	614	0.86	[0.20; 3.76]	1.0%
de Sanjosé 1994	36	249	26	242	1.40	[0.82; 2.41]	1.6%
de Sanjosé 1994	58	223	28	238	2.64	[1.61; 4.32]	1.7%
Graham 1982	83	134	56	130	2.15	[1.31; 3.52]	1.7%
Graham 1982	21	47	56	130	1.07	[0.55; 2.09]	1.5%
Janda 1973	9	18	3	18	5.00	[1.07; 23.46]	0.9%
Janda 1973	30	61	11	57	4.05	[1.77; 9.26]	1.4%
Kalimo 1981	4	7	0	26	68.14	[2.99; 1554.27]	0.4%
Kawana 1976	8	14	6	14	1.78	[0.40; 7.94]	0.9%
Kessler 1974	132	350	84	350	1.92	[1.38; 2.66]	1.8%
Kumar 1980	33	35	20	32	9.90	[2.01; 48.87]	0.9%
Lehtinen 1989	9	15	18	30	1.00	[0.28; 3.54]	1.1%
Lehtinen 1989	11	14	22	28	1.00	[0.21; 4.78]	0.9%
Lehtinen 1992	14	32	26	62	1.08	[0.46; 2.55]	1.4%
Lehtinen 1996	11	72	37	143	0.52	[0.25; 1.09]	1.5%
Lehtinen 2002	27	178	63	525	1.31	[0.81; 2.13]	1.7%
McDonald 1974	12	39	15	39	0.71	[0.28; 1.82]	1.3%
McDonald 1974	40	107	28	107	1.68	[0.94; 3.02]	1.6%
Mendis 1981	13	25	3	24	7.58	[1.79; 32.07]	1.0%
Mendis 1981	14	22	11	28	2.70	[0.85; 8.57]	1.2%
Mendis 1981	51	79	14	59	5.85	[2.75; 12.47]	1.5%
Muñoz 1995	75	234	58	213	1.26	[0.84; 1.90]	1.7%
Muñoz 1995	82	195	68	189	1.29	[0.86; 1.95]	1.7%
Nahmias 1974	37	57	12	82	10.79	[4.76; 24.48]	1.4%
Najem 1983	22	32	12	32	3.67	[1.30; 10.32]	1.3%
Pérez 2006	9	34	12	79	2.01	[0.76; 5.35]	1.3%
Pérez 2006	22	79	12	79	2.15	[0.98; 4.73]	1.5%
Pérez 2006	17	74	12	79	1.67	[0.73; 3.78]	1.4%
Skinner 1977	12	15	26	192	25.54	[6.75; 96.66]	1.1%
Thiry 1977	35	47	11	51	10.61	[4.16; 27.03]	1.3%
Thiry 1977	52	88	11	51	5.25	[2.38; 11.59]	1.5%
Vass−Sorensen 1984	11	44	5	30	1.67	[0.51; 5.41]	1.2%
Vestergaard 1972	115	135	54	115	6.50	[3.57; 11.83]	1.6%
Vonka 1984	14	76	30	205	1.32	[0.66; 2.65]	1.5%
Vonka 1984	2	21	30	205	0.61	[0.14; 2.77]	0.9%
Vonka 1984	19	145	31	267	1.15	[0.62; 2.11]	1.6%
Wilkie 1980	21	40	2	23	11.61	[2.40; 56.21]	0.9%
Wilkie 1980	23	29	2	23	40.25	[7.31; 221.68]	0.8%
**Random effects model**		4442		9659	2.23	[1.74; 2.85]	67.1%
Heterogeneity: I^2 = 74%, τ^2 = 0.5576, p < 0.01							
**Random effects model**		**7166**		**13314**	**2.56**	**[2.07; 3.15]**	**100.0%**
Heterogeneity: I^2 = 77%, τ^2 = 0.6232, p < 0.01							
Test for subgroup differences: χ^2_3 = 6.66, df = 3 (p = 0.08)							

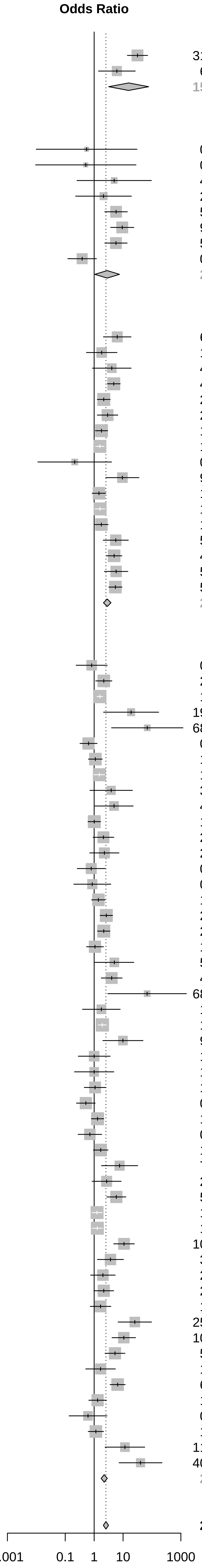

Supplementary Figure 6. EBV and cervical cancer (CC) or precancerous cervical lesions (PCL): subgroup meta-analyses forest plots according to different stages of disease (A), viral detection methods (B), different HDI regions (C), and specimen types (D)

A.

Study	PCL or CC Events	PCL or CC Total	Controls Events	Controls Total	OR	95%−CI	Weight
**Population = CIN 1**							
Ammatuna 2000	8	52	3	51	2.91	[0.73; 11.66]	3.1%
Aromseree 2015	25	85	11	82	2.69	[1.22; 5.91]	4.2%
Cameron 2018	14	50	40	266	2.20	[1.09; 4.44]	4.4%
Feng 2021	1	1	20	159	20.41	[0.80; 518.15]	1.2%
Khenchouche 2013	2	16	2	14	0.86	[0.10; 7.04]	2.1%
Kienka 2019	3	4	19	52	5.21	[0.51; 53.69]	1.9%
Landers 1993	0	25	0	25	1.00	[0.02; 52.36]	0.9%
Marinho−Dias 2013	2	21	3	18	0.53	[0.08; 3.56]	2.3%
McCormick 2015	13	17	8	15	2.84	[0.63; 12.89]	2.9%
Shoji 1997	3	44	0	20	3.46	[0.17; 70.15]	1.3%
Silver 2011	2	2	36	246	28.84	[1.36; 612.93]	1.3%
Szkaradkiewicz 2004	4	12	8	26	1.12	[0.26; 4.85]	3.0%
**Random effects model**		329		974	2.31	[1.54; 3.47]	28.7%
Heterogeneity: $I^2 = 0\%$, $\tau^2 = < 0.0001$, $p = 0.58$							
**Population = CIN 2/3**							
Ammatuna 2000	0	7	3	51	0.92	[0.04; 19.73]	1.3%
Aromseree 2015	42	85	11	82	6.30	[2.94; 13.54]	4.3%
Cameron 2018	9	14	40	266	10.17	[3.24; 31.92]	3.6%
Feng 2021	4	5	20	159	27.80	[2.96; 261.36]	2.0%
Khenchouche 2013	8	21	2	14	3.69	[0.65; 20.97]	2.6%
Kienka 2019	4	4	19	52	15.46	[0.79; 302.76]	1.3%
Landers 1993	4	50	0	25	4.94	[0.26; 95.38]	1.3%
Marinho−Dias 2013	1	22	3	18	0.24	[0.02; 2.52]	1.8%
McCormick 2015	7	15	8	15	0.77	[0.18; 3.21]	3.1%
Santos 2009	14	66	8	89	2.73	[1.07; 6.95]	4.0%
Seo 2005	3	20	0	20	8.20	[0.40; 169.90]	1.3%
Shoji 1997	15	70	0	20	11.45	[0.65; 200.21]	1.4%
Silver 2011	4	7	36	246	7.78	[1.67; 36.21]	2.9%
Szkaradkiewicz 2004	7	10	8	26	5.25	[1.07; 25.70]	2.8%
**Random effects model**		396		1083	4.32	[2.42; 7.70]	33.7%
Heterogeneity: $I^2 = 38\%$, $\tau^2 = 0.4051$, $p = 0.07$							
**Population = CC**							
Aromseree 2015	14	40	11	82	3.48	[1.40; 8.62]	4.0%
Kahla 2012	13	44	5	81	6.37	[2.09; 19.40]	3.6%
Khenchouche 2013	40	58	2	14	13.33	[2.70; 65.84]	2.8%
Landers 1993	5	18	0	25	20.78	[1.07; 404.70]	1.3%
Marinho−Dias 2013	2	9	3	18	1.43	[0.19; 10.57]	2.2%
McCormick 2015	11	18	8	15	1.38	[0.34; 5.51]	3.1%
Santos 2009	9	14	8	89	18.23	[4.91; 67.72]	3.3%
Sasagawa 2000	7	14	2	12	5.00	[0.79; 31.63]	2.4%
Sasagawa 2000	23	31	13	35	4.87	[1.69; 14.00]	3.7%
Se Thoe 1993	5	8	0	15	48.71	[2.15; 1101.85]	1.2%
Seo 2005	1	56	0	20	1.11	[0.04; 28.31]	1.2%
Shimakage 2001	14	16	0	4	52.20	[2.10; 1300.33]	1.2%
Shoji 1997	13	60	0	20	11.65	[0.66; 205.48]	1.4%
Silver 2011	3	4	36	246	17.50	[1.77; 172.92]	1.9%
Zhang 1992	11	80	19	43	0.20	[0.08; 0.48]	4.1%
**Random effects model**		470		719	4.89	[2.18; 10.96]	37.6%
Heterogeneity: $I^2 = 78\%$, $\tau^2 = 1.6055$, $p < 0.01$							
**Random effects model**		**1195**		**2776**	**3.69**	**[2.47; 5.50]**	**100.0%**

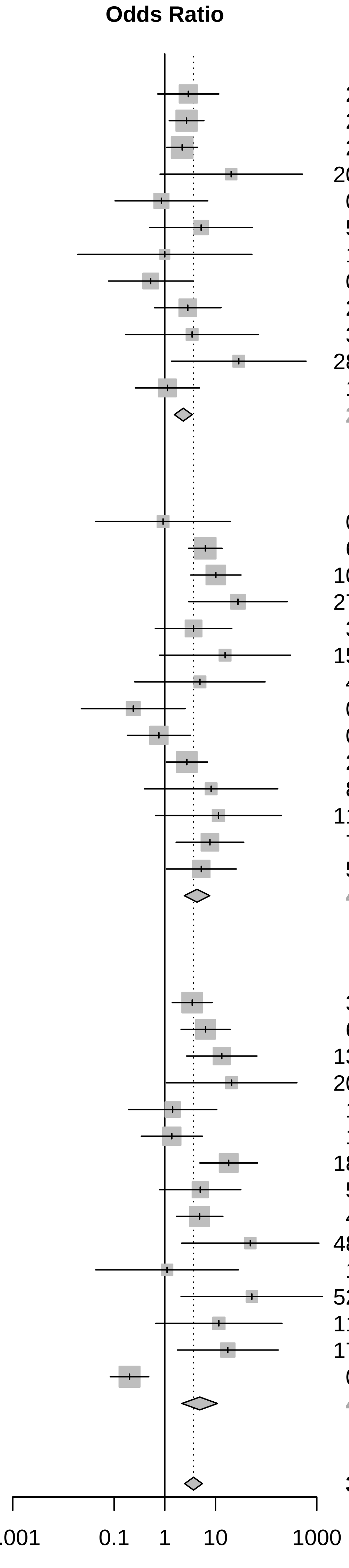

Heterogeneity: $I^2 = 60\%$, $\tau^2 = 0.8210$, $p < 0.01$

Test for subgroup differences: $\chi^2_2 = 4.51$, df = 2 ($p = 0.11$)

B.

Study	PCL or CC Events	PCL or CC Total	Controls Events	Controls Total	OR	95%−CI	Weight
**Method = PCR−based**							
Ammatuna 2000	8	52	3	51	2.91	[0.73; 11.66]	2.9%
Ammatuna 2000	0	7	3	51	0.92	[0.04; 19.73]	1.2%
Aromseree 2015	14	40	11	82	3.48	[1.40; 8.62]	3.7%
Aromseree 2015	25	85	11	82	2.69	[1.22; 5.91]	4.0%
Aromseree 2015	42	85	11	82	6.30	[2.94; 13.54]	4.0%
Cameron 2018	9	14	40	266	10.17	[3.24; 31.92]	3.3%
Cameron 2018	14	50	40	266	2.20	[1.09; 4.44]	4.1%
Feng 2021	4	5	20	159	27.80	[2.96; 261.36]	1.8%
Feng 2021	1	1	20	159	20.41	[0.80; 518.15]	1.1%
Kahla 2012	13	44	5	81	6.37	[2.09; 19.40]	3.4%
Khenchouche 2013	40	58	2	14	13.33	[2.70; 65.84]	2.6%
Khenchouche 2013	2	16	2	14	0.86	[0.10; 7.04]	1.9%
Khenchouche 2013	8	21	2	14	3.69	[0.65; 20.97]	2.4%
Kienka 2019	4	4	19	52	15.46	[0.79; 302.76]	1.2%
Kienka 2019	3	4	19	52	5.21	[0.51; 53.69]	1.7%
Marinho−Dias 2013	2	9	3	18	1.43	[0.19; 10.57]	2.0%
Marinho−Dias 2013	2	21	3	18	0.53	[0.08; 3.56]	2.2%
Marinho−Dias 2013	1	22	3	18	0.24	[0.02; 2.52]	1.7%
McCormick 2015	11	18	8	15	1.38	[0.34; 5.51]	2.9%
McCormick 2015	13	17	8	15	2.84	[0.63; 12.89]	2.7%
McCormick 2015	7	15	8	15	0.77	[0.18; 3.21]	2.8%
Santos 2009	9	14	8	89	18.23	[4.91; 67.72]	3.0%
Santos 2009	14	66	8	89	2.73	[1.07; 6.95]	3.7%
Sasagawa 2000	23	31	13	35	4.87	[1.69; 14.00]	3.5%
Sasagawa 2000	20	23	13	35	11.28	[2.80; 45.47]	2.9%
Seo 2005	1	56	0	20	1.11	[0.04; 28.31]	1.1%
Seo 2005	3	20	0	20	8.20	[0.40; 169.90]	1.2%
Shoji 1997	13	60	0	20	11.65	[0.66; 205.48]	1.3%
Shoji 1997	3	44	0	20	3.46	[0.17; 70.15]	1.2%
Shoji 1997	15	70	0	20	11.45	[0.65; 200.21]	1.3%
Silver 2011	2	2	36	246	28.84	[1.36; 612.93]	1.2%
Silver 2011	2	5	36	246	3.89	[0.63; 24.09]	2.3%
Silver 2011	2	2	36	246	28.84	[1.36; 612.93]	1.2%
Silver 2011	3	4	36	246	17.50	[1.77; 172.92]	1.7%
Szkaradkiewicz 2004	4	12	8	26	1.12	[0.26; 4.85]	2.8%
Szkaradkiewicz 2004	7	10	8	26	5.25	[1.07; 25.70]	2.6%
**Random effects model**		**1007**		**2908**	**3.92**	**[2.84; 5.41]**	**84.3%**
Heterogeneity: $I^2 = 36\%$, $\tau^2 = 0.2868$, $p = 0.02$							
**Method = Hybridization−based**							
Landers 1993	5	18	0	25	20.78	[1.07; 404.70]	1.2%
Landers 1993	0	25	0	25	1.00	[0.02; 52.36]	0.8%
Landers 1993	2	25	0	25	5.43	[0.25; 118.96]	1.1%
Landers 1993	2	25	0	25	5.43	[0.25; 118.96]	1.1%
Sasagawa 2000	7	14	2	12	5.00	[0.79; 31.63]	2.2%
Sasagawa 2000	6	17	2	12	2.73	[0.44; 16.75]	2.3%
Se Thoe 1993	5	8	0	15	48.71	[2.15; 1101.85]	1.1%
Shimakage 2001	14	16	0	4	52.20	[2.10; 1300.33]	1.1%
Shimakage 2001	4	5	0	4	27.00	[0.85; 856.53]	1.0%
Zhang 1992	11	80	19	43	0.20	[0.08; 0.48]	3.8%
**Random effects model**		**233**		**190**	**4.85**	**[1.32; 17.80]**	**15.7%**
Heterogeneity: $I^2 = 76\%$, $\tau^2 = 2.5445$, $p < 0.01$							
**Random effects model**		**1240**		**3098**	**3.89**	**[2.66; 5.67]**	**100.0%**
Heterogeneity: $I^2 = 57\%$, $\tau^2 = 0.7820$, $p < 0.01$							
Test for subgroup differences: $\chi^2_1 = 0.10$, df = 1 ($p = 0.76$)							

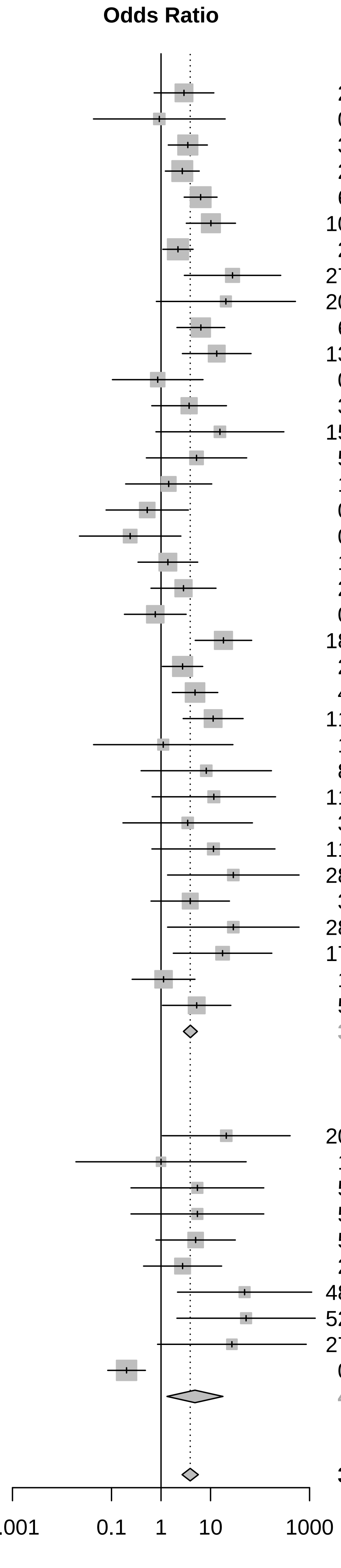

C.

Study	PCL or CC Events	PCL or CC Total	Controls Events	Controls Total	OR	95%−CI	Weight
**HDI = Medium HDI**							
Cameron 2018	9	14	40	266	10.17	[3.24; 31.92]	3.3%
Cameron 2018	14	50	40	266	2.20	[1.09; 4.44]	4.1%
Silver 2011	2	2	36	246	28.84	[1.36; 612.93]	1.2%
Silver 2011	2	5	36	246	3.89	[0.63; 24.09]	2.3%
Silver 2011	2	2	36	246	28.84	[1.36; 612.93]	1.2%
Silver 2011	3	4	36	246	17.50	[1.77; 172.92]	1.7%
**Random effects model**		**77**		**1516**	**6.70**	**[2.59; 17.28]**	**13.7%**
Heterogeneity: $I^2 = 51\%$, $\tau^2 = 0.6027$, $p = 0.07$							
**HDI = High HDI**							
Feng 2021	4	5	20	159	27.80	[2.96; 261.36]	1.8%
Feng 2021	1	1	20	159	20.41	[0.80; 518.15]	1.1%
Aromseree 2015	14	40	11	82	3.48	[1.40; 8.62]	3.7%
Aromseree 2015	25	85	11	82	2.69	[1.22; 5.91]	4.0%
Aromseree 2015	42	85	11	82	6.30	[2.94; 13.54]	4.0%
Kahla 2012	13	44	5	81	6.37	[2.09; 19.40]	3.4%
Khenchouche 2013	40	58	2	14	13.33	[2.70; 65.84]	2.6%
Khenchouche 2013	2	16	2	14	0.86	[0.10; 7.04]	1.9%
Khenchouche 2013	8	21	2	14	3.69	[0.65; 20.97]	2.4%
McCormick 2015	11	18	8	15	1.38	[0.34; 5.51]	2.9%
McCormick 2015	13	17	8	15	2.84	[0.63; 12.89]	2.7%
McCormick 2015	7	15	8	15	0.77	[0.18; 3.21]	2.8%
Santos 2009	9	14	8	89	18.23	[4.91; 67.72]	3.0%
Santos 2009	14	66	8	89	2.73	[1.07; 6.95]	3.7%
Se Thoe 1993	5	8	0	15	48.71	[2.15; 1101.85]	1.1%
Zhang 1992	11	80	19	43	0.20	[0.08; 0.48]	3.8%
**Random effects model**		**573**		**968**	**3.53**	**[1.80; 6.94]**	**44.9%**
Heterogeneity: $I^2 = 78\%$, $\tau^2 = 1.3101$, $p < 0.01$							
**HDI = Very high HDI**							
Ammatuna 2000	8	52	3	51	2.91	[0.73; 11.66]	2.9%
Ammatuna 2000	0	7	3	51	0.92	[0.04; 19.73]	1.2%
Kienka 2019	4	4	19	52	15.46	[0.79; 302.76]	1.2%
Kienka 2019	3	4	19	52	5.21	[0.51; 53.69]	1.7%
Landers 1993	5	18	0	25	20.78	[1.07; 404.70]	1.2%
Landers 1993	0	25	0	25	1.00	[0.02; 52.36]	0.8%
Landers 1993	2	25	0	25	5.43	[0.25; 118.96]	1.1%
Landers 1993	2	25	0	25	5.43	[0.25; 118.96]	1.1%
Marinho−Dias 2013	2	9	3	18	1.43	[0.19; 10.57]	2.0%
Marinho−Dias 2013	2	21	3	18	0.53	[0.08; 3.56]	2.2%
Marinho−Dias 2013	1	22	3	18	0.24	[0.02; 2.52]	1.7%
Sasagawa 2000	7	14	2	12	5.00	[0.79; 31.63]	2.2%
Sasagawa 2000	6	17	2	12	2.73	[0.44; 16.75]	2.3%
Sasagawa 2000	23	31	13	35	4.87	[1.69; 14.00]	3.5%
Sasagawa 2000	20	23	13	35	11.28	[2.80; 45.47]	2.9%
Seo 2005	1	56	0	20	1.11	[0.04; 28.31]	1.1%
Seo 2005	3	20	0	20	8.20	[0.40; 169.90]	1.2%
Shimakage 2001	14	16	0	4	52.20	[2.10; 1300.33]	1.1%
Shimakage 2001	4	5	0	4	27.00	[0.85; 856.53]	1.0%
Shoji 1997	13	60	0	20	11.65	[0.66; 205.48]	1.3%
Shoji 1997	3	44	0	20	3.46	[0.17; 70.15]	1.2%
Shoji 1997	15	70	0	20	11.45	[0.65; 200.21]	1.3%
Szkaradkiewicz 2004	4	12	8	26	1.12	[0.26; 4.85]	2.8%
Szkaradkiewicz 2004	7	10	8	26	5.25	[1.07; 25.70]	2.6%
**Random effects model**		**590**		**614**	**3.65**	**[2.30; 5.80]**	**41.4%**
Heterogeneity: $I^2 = 9\%$, $\tau^2 = 0.1449$, $p = 0.33$							
**Random effects model**		**1240**		**3098**	**3.89**	**[2.66; 5.67]**	**100.0%**

Heterogeneity: $I^2 = 57\%$, $\tau^2 = 0.7820$, $p < 0.01$

Test for subgroup differences: $\chi^2_2 = 1.41$, df = 2 ($p = 0.49$)

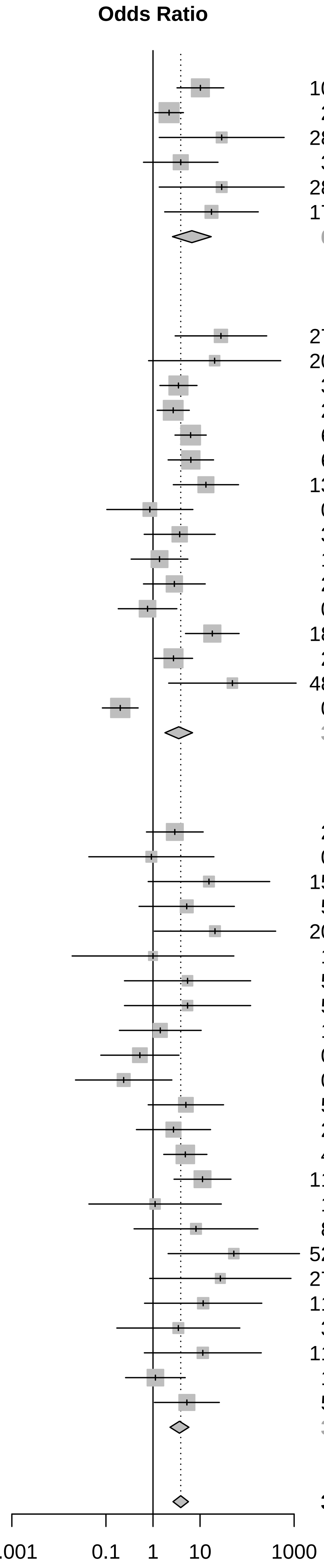

D.

Study	PCL or CC Events	PCL or CC Total	Controls Events	Controls Total	OR	95%−CI	Weight
**Specimen = Brush/swab**							
Ammatuna 2000	8	52	3	51	2.91	[0.73; 11.66]	3.4%
Ammatuna 2000	0	7	3	51	0.92	[0.04; 19.73]	1.4%
Cameron 2018	9	14	40	266	10.17	[3.24; 31.92]	3.8%
Cameron 2018	14	50	40	266	2.20	[1.09; 4.44]	4.7%
Feng 2021	4	5	20	159	27.80	[2.96; 261.36]	2.1%
Feng 2021	1	1	20	159	20.41	[0.80; 518.15]	1.3%
Kienka 2019	4	4	19	52	15.46	[0.79; 302.76]	1.5%
Kienka 2019	3	4	19	52	5.21	[0.51; 53.69]	2.0%
Marinho−Dias 2013	2	9	3	18	1.43	[0.19; 10.57]	2.4%
Marinho−Dias 2013	2	21	3	18	0.53	[0.08; 3.56]	2.5%
Marinho−Dias 2013	1	22	3	18	0.24	[0.02; 2.52]	2.0%
Santos 2009	9	14	8	89	18.23	[4.91; 67.72]	3.5%
Santos 2009	14	66	8	89	2.73	[1.07; 6.95]	4.2%
Seo 2005	1	56	0	20	1.11	[0.04; 28.31]	1.3%
Seo 2005	3	20	0	20	8.20	[0.40; 169.90]	1.4%
Silver 2011	2	2	36	246	28.84	[1.36; 612.93]	1.4%
Silver 2011	2	5	36	246	3.89	[0.63; 24.09]	2.7%
Silver 2011	2	2	36	246	28.84	[1.36; 612.93]	1.4%
Silver 2011	3	4	36	246	17.50	[1.77; 172.92]	2.1%
Szkaradkiewicz 2004	4	12	8	26	1.12	[0.26; 4.85]	3.3%
Szkaradkiewicz 2004	7	10	8	26	5.25	[1.07; 25.70]	3.0%
**Random effects model**		**380**		**2364**	**4.06**	**[2.37; 6.95]**	**51.5%**
Heterogeneity: $I^2 = 45\%$, $\tau^2 = 0.6104$, $p = 0.02$							
**Specimen = Biopsy(fresh−frozen)**							
Aromseree 2015	14	40	11	82	3.48	[1.40; 8.62]	4.3%
Aromseree 2015	25	85	11	82	2.69	[1.22; 5.91]	4.5%
Aromseree 2015	42	85	11	82	6.30	[2.94; 13.54]	4.6%
Kahla 2012	13	44	5	81	6.37	[2.09; 19.40]	3.9%
Khenchouche 2013	40	58	2	14	13.33	[2.70; 65.84]	3.0%
Khenchouche 2013	2	16	2	14	0.86	[0.10; 7.04]	2.3%
Khenchouche 2013	8	21	2	14	3.69	[0.65; 20.97]	2.8%
Se Thoe 1993	5	8	0	15	48.71	[2.15; 1101.85]	1.4%
Zhang 1992	11	80	19	43	0.20	[0.08; 0.48]	4.4%
**Random effects model**		**437**		**427**	**3.22**	**[1.24; 8.33]**	**31.1%**
Heterogeneity: $I^2 = 84\%$, $\tau^2 = 1.5899$, $p < 0.01$							
**Specimen = FFPE**							
Landers 1993	5	18	0	25	20.78	[1.07; 404.70]	1.5%
Landers 1993	0	25	0	25	1.00	[0.02; 52.36]	0.9%
Landers 1993	2	25	0	25	5.43	[0.25; 118.96]	1.4%
Landers 1993	2	25	0	25	5.43	[0.25; 118.96]	1.4%
Sasagawa 2000	7	14	2	12	5.00	[0.79; 31.63]	2.6%
Sasagawa 2000	6	17	2	12	2.73	[0.44; 16.75]	2.7%
Shimakage 2001	14	16	0	4	52.20	[2.10; 1300.33]	1.3%
Shimakage 2001	4	5	0	4	27.00	[0.85; 856.53]	1.2%
Shoji 1997	13	60	0	20	11.65	[0.66; 205.48]	1.5%
Shoji 1997	3	44	0	20	3.46	[0.17; 70.15]	1.4%
Shoji 1997	15	70	0	20	11.45	[0.65; 200.21]	1.5%
**Random effects model**		**319**		**192**	**6.61**	**[2.93; 14.87]**	**17.4%**
Heterogeneity: $I^2 = 0\%$, $\tau^2 = 0$, $p = 0.88$							
**Random effects model**		**1136**		**2983**	**4.13**	**[2.71; 6.29]**	**100.0%**
Heterogeneity: $I^2 = 58\%$, $\tau^2 = 0.8515$, $p < 0.01$							
Test for subgroup differences: $\chi^2_2 = 1.47$, df = 2 ($p = 0.48$)							

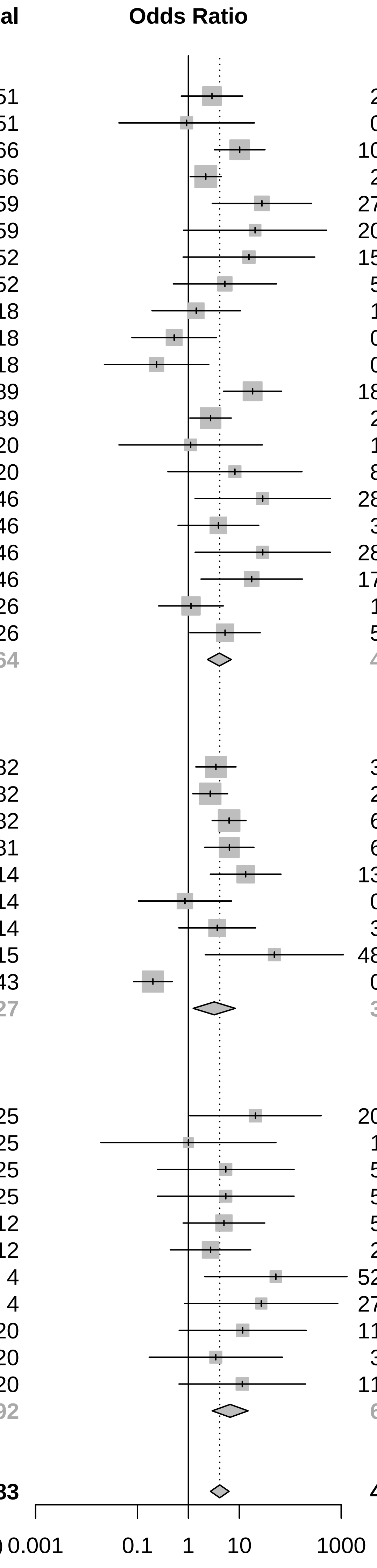

Supplement: Supplementary file 3 — Supplementary Material 3 [file 12985_2023_2234_MOESM3_ESM.pdf]
